# UNC79 and UNC80, Putative Auxiliary Subunits of the NARROW ABDOMEN Ion Channel, Are Indispensable for Robust Circadian Locomotor Rhythms in *Drosophila*

**DOI:** 10.1371/journal.pone.0078147

**Published:** 2013-11-05

**Authors:** Bridget C. Lear, Eric J. Darrah, Benjamin T. Aldrich, Senetibeb Gebre, Robert L. Scott, Howard A. Nash, Ravi Allada

**Affiliations:** 1 Department of Biology, University of Iowa, Iowa City, Iowa, United States of America; 2 Laboratory of Molecular Biology, National Institute of Mental Health, Bethesda, Maryland, United States of America; 3 Department of Neurobiology, Northwestern University, Evanston, Illinois, United States of America; University of Alabama at Birmingham, United States of America

## Abstract

In the fruit fly *Drosophila melanogaster,* a network of circadian pacemaker neurons drives daily rhythms in rest and activity. The ion channel NARROW ABDOMEN (NA), orthologous to the mammalian sodium leak channel NALCN, functions downstream of the molecular circadian clock in pacemaker neurons to promote behavioral rhythmicity. To better understand the function and regulation of the NA channel, we have characterized two putative auxiliary channel subunits in *Drosophila*, *unc79 (aka dunc79)* and *unc80 (aka CG18437).* We have generated novel *unc79* and *unc80* mutations that represent strong or complete loss-of-function alleles. These mutants display severe defects in circadian locomotor rhythmicity that are indistinguishable from *na* mutant phenotypes. Tissue-specific RNA interference and rescue analyses indicate that UNC79 and UNC80 likely function within pacemaker neurons, with similar anatomical requirements to NA. We observe an interdependent, post-transcriptional regulatory relationship among the three gene products, as loss of *na, unc79,* or *unc80* gene function leads to decreased expression of all three proteins, with minimal effect on transcript levels. Yet despite this relationship, we find that the requirement for *unc79* and *unc80* in circadian rhythmicity cannot be bypassed by increasing NA protein expression, nor can these putative auxiliary subunits substitute for each other. These data indicate functional requirements for UNC79 and UNC80 beyond promoting channel subunit expression. Immunoprecipitation experiments also confirm that UNC79 and UNC80 form a complex with NA in the *Drosophila* brain. Taken together, these data suggest that *Drosophila* NA, UNC79, and UNC80 function together in circadian clock neurons to promote rhythmic behavior.

## Introduction

Circadian rhythms are daily patterns of behavior and physiology driven by cellular clocks. Circadian clocks in metazoans consist of interdependent transcriptional feedback loops and post-translational modifications that produce ∼24 hour molecular oscillations. At the core of the *Drosophila* circadian clock, the transcription factor partners CLOCK (CLK) and CYCLE (CYC) upregulate the expression of *period (per)* and *timeless (tim).* PER and TIM proteins accumulate in the cytoplasm and later translocate to the nucleus, where they inhibit CLK-CYC activity and their own expression. This mechanism and others result in ∼24 hour rhythms in CLK-CYC transcription factor activity and in PER/TIM expression. This molecular clock is highly conserved in animals, and homologs of several *Drosophila* clock genes exhibit similar functions in mammals [1]. In *Drosophila,* the molecular clocks essential for daily activity rhythms are found in roughly 150 pacemaker neurons in the adult brain, and specific groups of these neurons have been shown to be important for different aspects of behavioral rhythmicity. A subset of pacemaker neurons express the neuropeptide Pigment-Dispersing Factor (PDF), and the PDF+ cells communicate to a broader group of pacemaker neurons to synchronize and enhance molecular clock oscillations [2].

An important component of circadian pacemaker neuronal output in *Drosophila* is NARROW ABDOMEN (NA), a putative sodium leak channel orthologous to mammalian NALCN. *Drosophila na* mutants exhibit strong defects in circadian locomotor behavior, as well as increased anesthetic sensitivity and “hesitant” walking [3]. Despite disruptions in circadian behavior, oscillations of the clock protein PER remain essentially intact in *na* mutants, indicating function primarily downstream of the molecular clock [4]. NA protein is expressed broadly in the adult *Drosophila* brain, and gene expression likely includes multiple groups of pacemaker neurons [3], [4]. Tissue-specific expression of *na* in most or all pacemaker neurons using the GAL4/ UAS system fully rescues rhythmic behavior in *na* mutants. Moreover, rescue of *na* rhythmicity phenotypes using the pan-neuronal driver *elavGAL4* is blocked when the GAL4 inhibitor GAL80 is expressed specifically in circadian neurons (*cryptochromeGAL80*) [4]. In mammals, electrophysiological characterization has demonstrated that NALCN functions as a voltage-insensitive, non-selective cation channel that contributes sodium leak conductance. NALCN knockout mice display severe defects in respiratory rhythm and die within a day of birth, and mutant hippocampal neurons exhibit hyperpolarized resting membrane potential [5].

Two putative auxiliary channel subunits for NA/NALCN, known as UNC79 and UNC80, were first identified in *C. elegans*, where mutants display anesthetic sensitivity phenotypes that resemble *Drosophila na* mutants [6]. Like NA/NALCN, UNC79 and UNC80 orthologs are found in all animals. These large proteins (> =  300 Kd in most animals) are unrelated to each other and have no clear functional domains [7], [8]. In *C. elegans,* loss of function of any of the putative subunits (*unc-79, unc-80,* or the NA/NALCN homologs *nca-1/2)* produces similar phenotypes, including defects in crawling and swimming behavior as well as anesthetic sensitivity [7], [9]. In *Drosophila*, mutation of the UNC79 ortholog (*unc79/ dunc79*) causes anesthetic sensitivity and hesitant walking phenotypes [7], while the *Drosophila* UNC80 ortholog (*CG18437)* has not been characterized. In *C. elegans,* the three putative subunits exhibit interdependence, as loss of function of one of these genes is associated with either decreased expression or disrupted localization of each of the corresponding proteins. This regulatory relationship may be post-transcriptional, as *nca-1* transcript levels are unaffected in either *unc-80* or *unc-79* mutants [7], [10]. Notably, *Drosophila unc79* mutants also express decreased levels of NA protein but not transcript, suggesting a possible conserved regulatory relationship [7]. Yet some differences in this relationship are evident in mammals, where *UNC79* mutant mice lack detectable levels of UNC80 protein but retain NALCN [11], [12].

Data from mammals has provided further insight into the function and regulation of this subfamily of ion channels. Co-immunoprecipitation experiments demonstrate that NALCN, UNC79 and UNC80 proteins form a complex in the mouse brain [11], [13]. Moreover, electrophysiological data indicate that NALCN channel activity is regulated through multiple G-protein coupled receptor (GPCR) signaling pathways. The substance P receptor TACR1 increases channel activity in a G-protein independent manner; this activation requires the UNC80 subunit and SRC kinase activity [13], [14]. The M3 muscarinic receptor may activate NALCN in pancreatic islet cells in a similar manner [15]. In contrast, the calcium sensing receptor (CaSR) GPCR appears to provide a tonic inhibition of NALCN channel activity that is G-protein dependent [11]. Notably, CaSR-mediated inhibition is disrupted in hippocampal neurons isolated from *UNC79* knockout mice, which express NALCN protein but not UNC80. Inhibition can be restored by transfection of UNC80 into *UNC79* mutant neurons, suggesting that the requirement for UNC79 in channel regulation can be bypassed by UNC80 [11].

Here we show that both UNC79 and UNC80 are important for promoting circadian behavioral rhythmicity in *Drosophila.* Similar to *C. elegans,* the three putative channel subunits in *Drosophila* exhibit an interdependent regulatory relationship that is primarily post-transcriptional. The anatomical requirements for all three gene products in circadian rhythms are similar or identical, and co-immunoprecipitation experiments demonstrate that these proteins form a complex in the *Drosophila* head. Transgenic expression of *unc79* or *unc80* in pacemaker neurons can restore rhythmicity to the corresponding mutant strain. However, transgenic expression of one subunit does not restore circadian behavior in the absence of another. Thus, both UNC79 and UNC80 likely have functional requirements in *Drosophila* beyond promoting expression of the other channel subunits. These requirements may include the modulation of NA channel activity, which may serve as an important mechanism for controlling the activity of circadian pacemaker neurons.

## Materials and Methods

### Drosophila strains

The *unc79^x25^* allele was generated by imprecise excision of P-element insertion *GS9462 (Drosophila* Genetics Resource Center). Genomic analysis indicates that *unc79^x25^* contains a 1305 bp genomic deletion in *unc79*, while retaining 12 bp of the P-element (**Figure S1A**). Transcript analysis was performed by cloning reverse transcriptase PCR (RT-PCR) products from *unc79^x25^/ Df(3R)ED5942* and *+/ Df(3R)ED5942* adult heads (TRIzol reagent, Superscript III Reverse Transcriptase, TOPO TA cloning; Invitrogen). All clones isolated from *unc79^x25^/ Df(3R)ED5942* (11/11) lacked the 57 bp and 80 bp exons corresponding to the deleted sequence (**Figure S1A**, exons 12–13); some of these clones (3/11) contained a 16 bp exon in lieu of the 57/80 bp exons. Conceptual translation confirms that all 11 *unc79^x25^* clones produce a shift in reading frame compared to the predicted wild-type UNC79 protein. The *unc80^x42^* allele was produced by imprecise excision of *GS12792 (Drosophila* Genetics Resource Center), an insertion into a predicted *unc80* coding exon (**Figure S1B**, exon 18). Genomic sequence analysis indicates that *unc80^x42^* retains 14 bp of *GS12792*, but lacks the UAS repeat sequence. Conceptual translation of *unc80^x42^* indicates that the 14 bp insertion introduces 2 stop codons and shifts the reading frame of any read-through products. Transgenic expression strains were produced by cloning *unc79* or *unc80* cDNAs into pUAST [16], followed by injection into *Drosophila* embryos (Bestgene Inc). Full-length *unc79* cDNA was subcloned from two partially overlapping cDNAs (RE64326, GH05210), and a 6X MYC tag was added at the C-terminus. Full-length *unc80* cDNA was generated by RT-PCR from wild-type head extracts (TRIzol reagent, Superscript III Reverse Transcriptase, TOPO TA cloning; Invitrogen), and a 3X HA tag was added at the N-terminus. For UAS-*unc79MYC,* two independent insertions (designated 23 and 24) were recombined on chromosome II to increase expression. All RNA interference (RNAi) strains and relevant controls were obtained from the Vienna *Drosophila* RNAi Center (VDRC) [17]. The *unc79* and *unc80* deficiency strains were obtained from the Bloomington *Drosophila* stock center [18], [19]. Other strains have been previously described: *pdfGAL4* [20], *timGAL4* [21], *elavGAL4* [22], UAS*-dcr2* [17], UAS*-na U3* [4], *na^har^* [3], *w^1118^ iso31* [19], *na^e04385^, unc79^f03453^,* and *unc79^f01615^* [7]. For the experiments indicated, *na, unc79,* and *unc80* mutant strains were backcrossed to *w^1118^ iso31* for 6-8 generations. After the final backcross, stable lines were generated from both wild-type and mutant individuals.

### Cross schemes and behavior analyses

*Drosophila* crosses were maintained at 25°C in 12 hr Light: 12 hr Dark (LD) conditions, except some *elavGAL4* UAS*-dicer2* X RNAi crosses were raised at room temperature to increase viability. To produce *unc79* and *unc80* homozygous mutant progeny, most mutant strains were maintained over the *TM3 hs-hid* balancer [23]. These crosses (plus controls) were heat-shocked for 1-2 hrs at 37°C after 4–6 days in order to kill *TM3 hs-hid* progeny. The *TM3 hs-hid* balancer was also backcrossed to *w^1118^ iso31* for 8 generations for use in balancing backcrossed *unc79* and *unc80* mutant and control strains.

For behavior analyses, locomotor activity levels were assayed from 0-7 day old adults for 5 days LD conditions followed by 7 days constant darkness (DD) at 25°C using the *Drosophila* Activity Monitor system (Trikinetics). The behavior data presented were analyzed from male flies, except where indicated. To produce LD activity profiles, activity levels of individual flies were normalized and averaged within genotypes over the last four days of LD conditions. Morning anticipation index (MAI) and evening anticipation index (EAI) were calculated from LD data by determining the largest 2–3 hour increase in normalized average activity of each genotype over the last 5 hours of dark phase (MAI) or the last 5 hours of light phase (EAI; modified from [4]). For DD analyses, chi-squared periodogram measurements were performed on individual flies using ClockLab analysis software (Actimetrics). Flies were considered rhythmic if the chi-squared power was > =  10 above significance, as previously described [4].

### Antibodies and Western blot

Anti-UNC79 and UNC80 sera were produced by expressing His-tagged polypeptides of either *unc79* (aa1843-1992, NP_001163652.1) or *unc80* (aa 3092-3187, NP_651577.3) from BL21(DE3) cells using the pET28 plasmid and His-Bind column purification system (EMD Millipore). Unconjugated protein was used to inject rabbits, sera were tested by ELISA, and terminal bleeds used for protein detection. Rabbit anti-NA was described previously [3]. Mouse anti-α-spectrin (3A9; [24]) was obtained from the Developmental Studies Hybridoma Bank (University of Iowa, Department of Biology). Western blots were performed as previously described, using adult head extracts from mixed light phase (ZT 0–10) samples [4]. For experiments in which protein levels were compared between genotypes, additional steps were taken to improve the reliability of quantitation [25]. Equal amounts of head extract (4–10 µg, depending on experiment) were loaded in each lane as determined by Bradford protein assay (Bio-Rad), and similar protein levels were confirmed after transfer using SYPRO Ruby Protein Blot staining (Lonza) or Novex Reversible Membrane Protein Stain (Invitrogen). A minimum of three biological replicates was performed for each comparison made, and lane order was varied between experiments to control for uneven transfer. Protein levels were measured using NIH ImageJ gel analysis (http://rsbweb.nih.gov/ij/). To account for intensity differences based on exposure, protein levels were normalized to the average intensity of all samples within the blot; in most cases identical samples were loaded in each biological replicate. For NA, UNC79, and UNC80, the antisera used recognize both transgenic and endogenous proteins. Transgenic NA protein (derived from UAS-*na*) migrates more quickly on Western blot than the endogenous doublet, perhaps due to the use of a different start methionine [4]. For UNC79, a prominent band was often detected just below the endogenous and transgenic bands, but our evidence indicates that this band is non-specific. The intensity of this band varies depending on strain, rearing conditions, and immunoblotting procedures, and it is generally much more prominent when PVDF membrane is used as compared to nitrocellulose. Levels of this band are not consistently decreased using an RNAi strain that targets *unc79* transcript near the region of the anti-UNC79 antigen (VDRC *108132;* data not shown). Moreover, RNA-seq analyses have not identified any *unc79* splice variants likely to produce a detectable protein of this size in both *unc79^+^* and *unc79^x25^* strains (*Drosophila* modENCODE project).

### Co-immunoprecipitation

Protein was extracted from adult heads using homogenization buffer (0.25M sucrose, 10 mM Tris pH 7.4, 1 mM EDTA) with protease inhibitors (Roche). Nuclei and cellular debris were pelleted at 1000 x g for 10 min and the protein concentration of the cleared supernatant was measured using Bradford reagent (Bio-Rad). Supernatents were then subject to a second spin at 48,000 x g for 40 min in order to pellet membranes. This membrane pellet was resuspended in 150 mM NaCl, 50 mM Tris pH 7.4, 1 mM EDTA plus protease inhibitors. Incubation of resuspended membranes at a concentration of 2 mg/ml in 1% n-Dodecyl-β-D-Maltopyranoside, 1% Anapoe-58 (Affymetrix) in resuspension buffer for 40 minutes on ice was followed by a 20 minute spin at 133,000 x g (Beckman Coulter Airfuge) to pellet unsolubilized particles. Solubilized protein (80 µl) was incubated with 2 µl rabbit or mouse antiserum in a total volume of 0.5 ml and rocked at room temperature for one hour. A 2% bed volume of Protein-A:Sepharose beads (GE Healthcare) were added and the tube was rocked for one hour at 4°C. Beads were pelleted with a short spin, supernatant removed, and beads washed 3x in resuspension buffer with the above detergents. Elution was with 1/10^th^ volume LDS running buffer with reducing agent (Invitrogen) and 20 µl was loaded per gel lane.

### Statistical analysis

LD anticipation index values (MAI/EAI) and DD period values were compared among genotypes by Kruskal-Wallis one-way ANOVA followed by Dunn’s *post-hoc* test, using Sigmaplot (Systat Software). For DD rhythmicity data, the proportion of rhythmic flies was determined as described above, and comparisons were made between genotypes using Fisher’s exact test (Sigmaplot or Graphpad Quickcalcs, http://www.graphpad.com/quickcalcs/). For protein and RNA expression comparisons, significance was determined using Student’s t-test (Microsoft Excel).

## Results

### Drosophila unc79 and unc80 are required for robust behavioral rhythmicity

**Figure 1:**
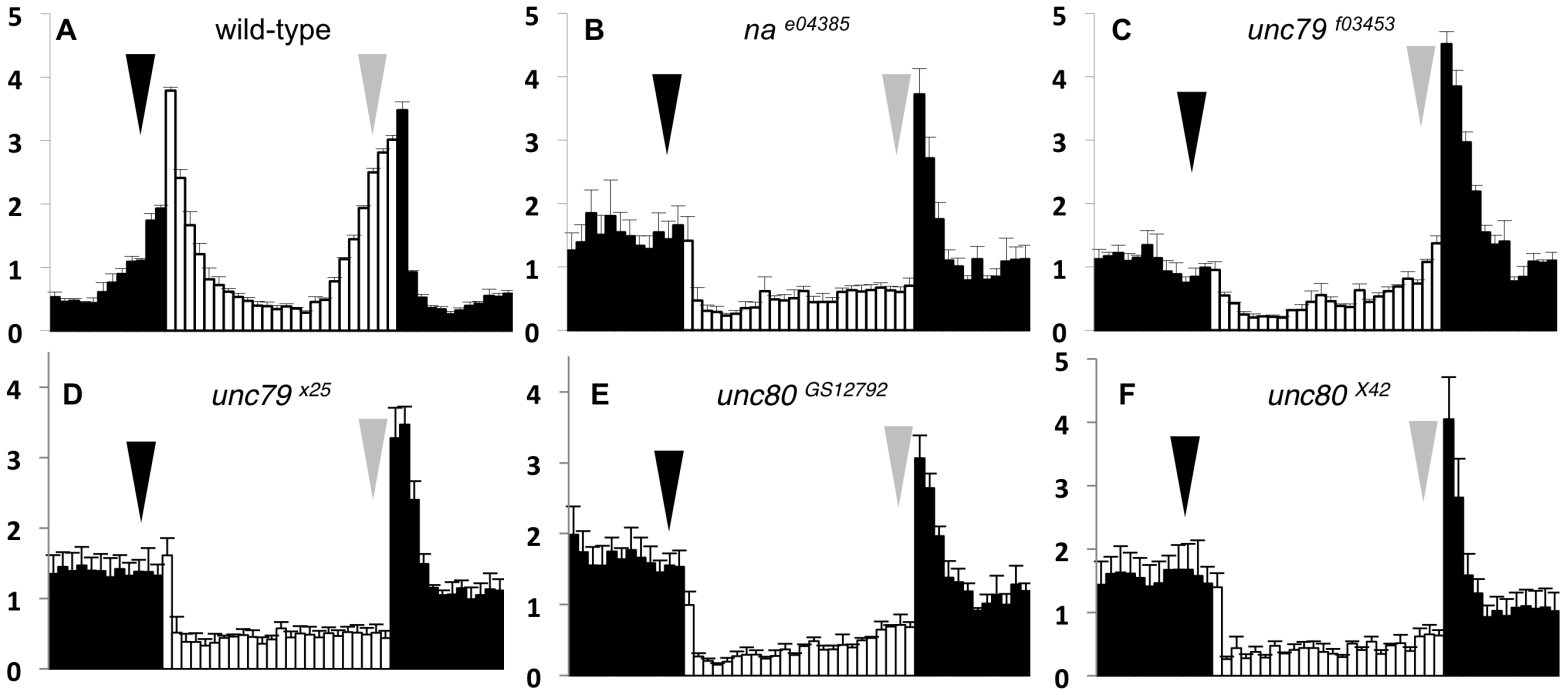
*Drosophila unc79* and *unc80* mutants display defects in anticipatory behavior. (A-F) Normalized locomotor activity profiles from adult male populations, averaged over four days of 12 hr light: 12 hr dark (LD) entrainment conditions. White bars indicate light phase; black bars indicate dark phase. Error bars represent standard error of the mean. Arrows indicate morning anticipation (black) and evening anticipation (gray). All strains were backcrossed to *w^1118^ (iso31)* for 6–8 generations. Morning anticipation index (MAI) and evening anticipation index (EAI) are indicated for each genotype (see Materials and Methods). (A) Representative wild-type strain (n = 52), MAI  =  1.5+/−0.1, EAI  =  2.1+/−0.1; (B) *na^e04385^* (n = 75), MAI  =  0.8+/−0.2, EAI  =  0.5+/−0.1; (C) *unc79^f03453^* (n = 44), MAI  =  0.5+/−0.1, EAI  =  1.0+/−0.1; (D) *unc79^x25^* (n = 93), MAI  =  0.7+/−0.1, EAI  =  0.4+/−0.1; (E) *unc80^GS12792^* (n = 64), MAI  =  0.7+/−0.2, EAI  =  0.5+/−0.1; (F) *unc80^x42^* (n = 67), MAI  =  0.9+/−0.2, EAI  =  0.5+/−0.1. Comparison of MAI values using Kruskal-Wallis one-way ANOVA indicates a significant difference among groups (P<0.001, 5 degrees of freedom), with each of the mutant genotypes (B-F) differing from the wild-type strain (A; Dunn’s method, P<0.05). Significant differences among genotypes are also observed for EAI values (Kruskal-Wallis one-way ANOVA, P<0.001, 5 degrees of freedom). Each mutant strain again differs from wild-type, and *unc79^f03453^* (C) also differs significantly from the other mutant genotypes (B, D-F; Dunn’s method, P<0.05). No significant differences in MAI or EAI are observed among the strong mutant alleles *na^e04385^* (B), *unc79^x25^* (D), *unc80^GS12792^* (E) or *unc80^x42^* (F), as determined by Dunn’s method.

*Drosophila unc79* and *CG18437* (herein referred to as *unc80)* encode putative auxiliary subunits of the NA ion channel. We have previously shown that the pore-forming subunit NA is an important component of circadian pacemaker neuronal output [4]. To determine whether *unc79* and *unc80* also contribute to circadian rhythmicity, we assessed locomotor activity rhythms in strains mutated for either gene. An intronic insertion allele of *unc79, unc79^f03453^* (**Figure S1A**), was previously shown to exhibit anesthetic sensitivity phenotypes similar to loss of *na* [7]. We observe that *unc79^f03453^* mutants exhibit decreased morning and evening anticipatory behavior during 12 hr light: 12 hr dark conditions (LD), similar to *na* mutants (**Figure 1A-C**, arrows). Yet the evening anticipation phenotype observed in *unc79^f03453^* mutants (EAI  =  1.0+/−0.1) is less severe than in *na^e04385^* mutants (**Figure 1B-C**, gray arrows; EAI  =  0.5+/−0.1, P<0.05). Furthermore, *unc79^f03453^* flies exhibit only a moderate loss of rhythmicity in constant dark conditions (DD) relative to a wild-type control strain (P  =  0.015); this phenotype is also less severe than that of *na* mutants (*na^har^, na^e04385^*) maintained in the same genetic background (**Table 1**, P<0.001). Evaluation of *unc79^f03453^* by RT-PCR suggests that some wild-type transcript is produced in this strain (data not shown), indicating that this allele is likely hypomorphic. Therefore, we used transposon excision to generate a novel *unc79* mutant strain. This strain, *unc79^x25^,* contains a ∼1300 bp genomic deletion that encompasses two putative coding exons (**Figure S1A**). Sequencing of RT-PCR products derived from *unc79^x25^* reveals that a 57 bp exon and a 80 bp exon are always absent, and all products isolated contain a shift in the predicted reading frame (see *Materials and Methods).* We find that *unc79^x25^* mutants exhibit severe defects in both anticipatory behavior (**Figure 1D**, P<0.05) and free-running rhythmicity (**Table 1**, P<0.001) in comparison to wild-type flies; these behavioral phenotypes are indistinguishable from strong loss-of-function alleles of *na* (P  =  1.00 for DD rhythmicity). All of the mutants assayed were backcrossed to an isogenic *w^1118^* strain (*iso31)* for six to eight generations, minimizing the influence of genetic background differences on the observed phenotypes (**Figure 1** **,** **Table 1**). Notably, *unc79^x25^* mutants exhibit similarly strong phenotypes when assayed in trans to a chromosomal deficiency (Df) that deletes the *unc79* locus (*Df(3R) ED5942*; **Table S1**, P  =  1.00 and data not shown). Thus, *unc79^x25^* likely represents a complete or severe loss of *unc79* gene function.

**Table 1:** *unc79* and *unc80* mutants exhibit severe defects in free-running rhythmicity.

Genotype 1	Period (hrs)	Power	Rhythmic (%)	n
*na [har+]*	23.9+/−0.1	66+/−6	97	29
*na [har]*	NA	1+/−0	0	19
*na [e04385+]*	24.1+/−0.1	81+/−5	98	50
*na[e04385]*	31.5 2	1+/−0	3	33
*unc79 [f03453+]*	23.9+/−0.0	74+/−6	96	52
*unc79 [f03453]*	24.6+/−0.2 2	40+/−8	75	20
*unc79 [x25+]*	23.9+/−0.1	65+/−7	95	41
*unc79 [x25]*	33.5 2	1+/−1	3	31
*unc80 [GS12792+]*	23.9+/−0.0	81+/−4	99	84
*unc80 [GS12792]*	22.0 2	1+/−0	4	27
*unc80 [x42+]*	23.8+/−0.1	53+/−6	79	48
*unc80 [x42]* 3	NA	0+/−0	0	9

1 All strains were backcrossed to *iso31* for > = 6 generations, and stable lines were established from both mutant and wild-type (+) individuals.

2 Period length in *unc79 [f03453]* differs significantly from wild-type (Kruskal-Wallis one-way ANOVA, 1 degree of freedom, P<0.001). Period measurements in *na[e04385], unc79[x25],* and *unc80[GS12792]* are based on single weakly rhythmic flies, and were thus excluded from statistical analysis.

3 Few flies survived to the end of DD; refer to Figure 1F for LD phenotype.

For *unc80,* we assayed circadian behavior in a strain that contains a transposon insertion in a putative coding exon, *unc80^GS12792^* (**Figure S1B**). *unc80^GS12792^* mutants exhibit strong defects in both anticipatory behavior (**Figure 1E**, P<0.05) and free-running rhythmicity (**Table 1**, P<0.001) when compared to wild-type controls. We generated an additional *unc80* allele by incomplete excision of the *GS12792* P-element insertion. This strain, *unc80^x42^*, retains 14 bp of the transposon insertion but lacks a UAS element present in *unc80^GS12792^* (see *Materials and Methods*). Like *unc80^GS12792^, unc80^x42^* mutants exhibit severe disruptions in circadian behavior (**Figure 1F**, P<0.05; **Table 1**, P<0.001). To confirm that the observed phenotypes map to the *unc80* locus, we generated *unc80^GS12792^/Df* and *unc80^x42^/Df* trans-heterozygotes; these flies exhibit phenotypes similar to homozygous *unc80^GS12792^* or *unc80^x42^* mutants (**Table S1**, P  =  1.00; data not shown). Both alleles were also backcrossed for eight generations to the *iso31* strain. All strains that retain an *unc80* insertion element exhibit a similar, strong circadian phenotype while all *unc80+* backcrossed strains exhibit normal rhythmicity (compiled in **Figure 1** and **Table 1**, data not shown). Thus, our data indicate that *unc80^GS12792^* and *unc80^x42^* retain little or no *unc80* function. For both *unc79* and *unc80,* strong mutant alleles exhibit circadian behavioral phenotypes that are indistinguishable from *na* mutants, suggesting that both genes may be required for *na* function.

To assess potential genetic interactions among *na, unc79,* and *unc80,* we also examined circadian behavior in single versus double heterozygotes. Neither *unc79^x25^/+* nor *unc79^x25^/ unc80^42^* males exhibit significant DD rhythmicity defects when compared to wild-type controls (**Table S2**, P > =  0.082), although a subtle decrease in rhythmicity is observed in *unc80^x42^/*+ single heterozygotes (**Table S2**, P  =  0.049). As the *na* locus is on the X chromosome, we assayed *na*/+ heterozygous combinations in females. Neither *na^e04385^/+;; unc79^x25^/+* nor *na^e04385^/+;; unc80^42^/+* flies exhibit strong deficits in DD rhythmicity compared to single heterozygotes (**Table S2**, P > =  0.380). However, baseline rhythmicity of females in this genetic background *(iso31)* is not very robust, so subtle defects might be difficult to assess.

### UNC79, UNC80, and NA have similar anatomical requirements

**Figure 2:**
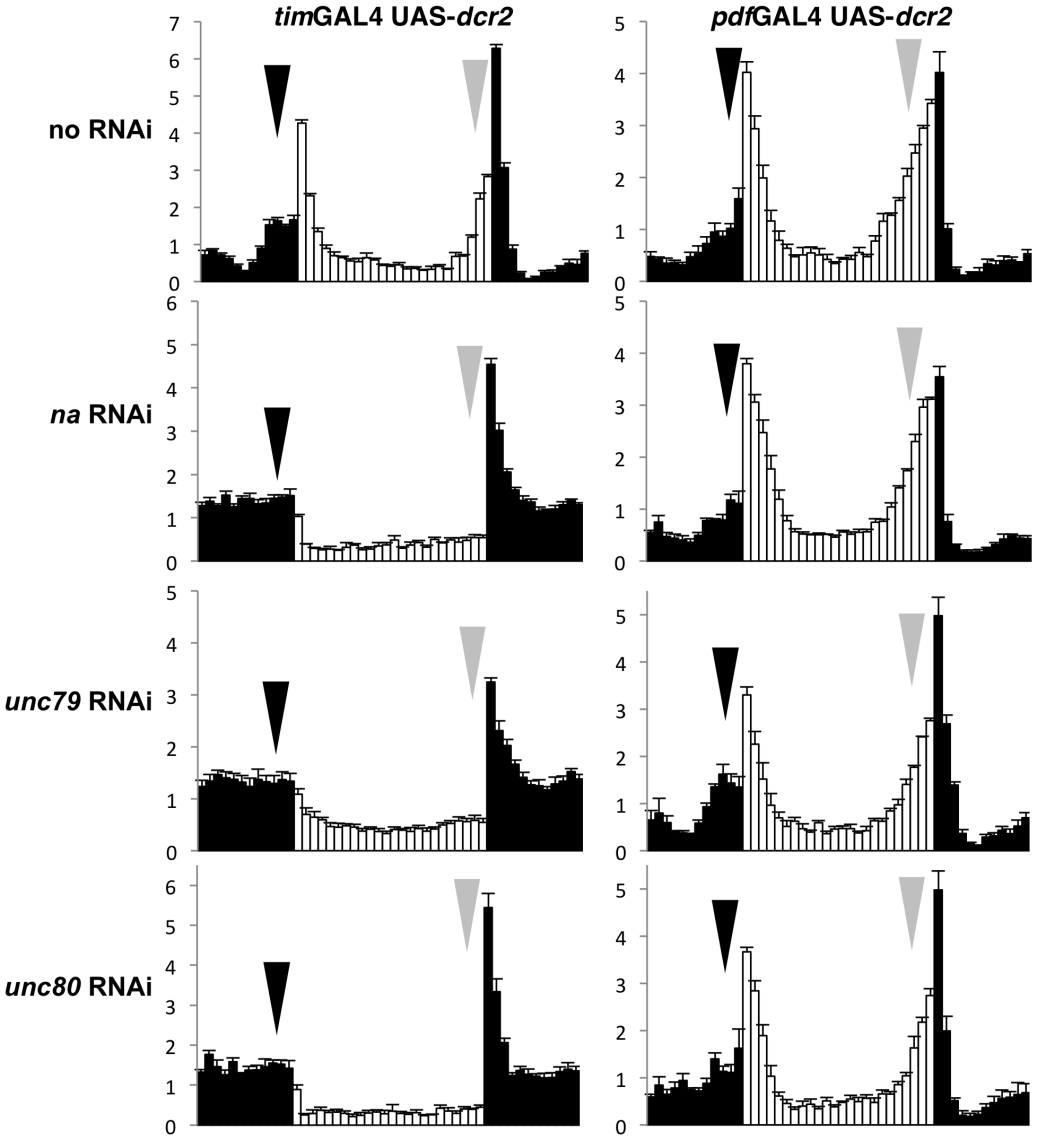
RNAi knockdown of *na, unc79*, or *unc80* in all pacemaker neurons results in anticipation defects. Normalized activity profiles from adult male populations averaged over four days of LD entrainment. White bars represent light phase; black bars indicate dark phase. Error bars represent standard error of the mean. Arrows indicate morning anticipation (black) and evening anticipation (gray). The genotypes represented in the left panels are *timGAL4*/ +; UAS-*dcr2*/ +, heterozygous for the following insertions from the Vienna *Drosophila* RNAi Center (VDRC): (top panel) no RNAi  =  control strain *attp VIE-260B* (n =  55), MAI  =  1.5+/−0.1, EAI  =  2.6+/−0.1; (second panel) *na = * 103754 (n  =  43), MAI  =  0.5+/−0.1, EAI  =  0.4+/−0.1; (third panel) *unc79  = * 108132 (n =  44), MAI  =  0.3+/−0.1, EAI  =  0.3+/−0.1; (bottom panel) *unc80  = * 108934 (n =  42), MAI  =  0.7+/−0.1, EAI  =  0.3+/−0.1. Genotypes represented in the right panels are *pdfGAL4* UAS-*dcr2*/+ heterozygous for the same VDRC strains: (top panel) *attp VIE-260B* (n =  32), MAI  =  1.3+/−0.1, EAI  =  2.4+/−0.2; (second panel) *na = * 103754 (n  =  33), MAI  =  1.0+/−0.1, EAI  =  2.3+/−0.2; (third panel) *unc79  = * 108132 (n =  37), MAI  =  1.5+/−0.2, EAI  =  2.1+/−0.2; (bottom panel) *unc80  = * 108934 (n =  35), MAI  =  1.3+/−0.2, EAI  =  2.3+/−0.2. Both MAI and EAI differ significantly among the *timGAL4/ +*; UAS-*dcr2/* + genotypes (Kruskal-Wallis one-way ANOVA, 3 degrees of freedom, P<0.001), and each RNAi genotype exhibits significantly lower MAI and EAI than the control strain (Dunn’s method, P<0.05). MAI and EAI values for each *timGAL4* UAS-*dcr2* RNAi strain are either lower or not significantly different from values calculated from strong mutant alleles for the corresponding gene (Kruskal-Wallis one-way ANOVA, 1-2 degrees of freedom). No significant differences in MAI or EAI are observed among *pdfGAL4* UAS-*dcr2*/ + genotypes (Kruskal-Wallis one-way ANOVA, 3 degrees of freedom, P > =  0.146).

To determine the anatomical requirements for *unc79* and *unc80* in circadian behavior, we utilized tissue-specific RNA interference (RNAi). We find that pan-neuronal knockdown of *na, unc79,* or *unc80* using *elavGAL4* driven expression of UAS-RNAi constructs results in a strong decrease in protein expression for the targeted gene, particularly when these constructs are co-expressed with the RNAi component *dicer2* (UAS-*dcr2*; **Figure S2A**). To address whether *unc79* and *unc80* function is required in pacemaker neurons, we crossed these UAS-RNAi lines to circadian GAL4 strains. Expression of *unc79, unc80,* or *na* RNAi using the broad circadian driver *timelessGAL4 (timGAL4)* plus UAS*-dcr2* produces strong LD and DD rhythmicity defects (**Figure 2**, left panels, P<0.05; **Table 2**, P<0.001), which are comparable to the strongest phenotypes observed in the corresponding mutant strains (**Figure 1**; **Table 1**, P > =  0.385). Restricting RNAi expression to the PDF+ pacemaker neurons using *pdfGAL4* UAS-*dcr2* produces strong defects in DD rhythmicity compared to control strains (**Table 2**, P<0.001), but does not clearly alter LD behavior (**Figure 2**, right panels, P > =  0.146). These data suggest that both *unc79* and *unc80* are required broadly among pacemaker neurons to promote locomotor rhythmicity. Moreover, both genes appear to have similar anatomical requirements as *na* itself.

**Table 2:** RNAi knockdown of *na, unc79,* or *unc80* in circadian pacemaker neurons decreases DD rhythmicity.

Genotype	Period (hrs)	Power	Rhythmic (%)	n
*UAS-naRNAi[103754]/+; UAS-dcr2/ +*	23.9+/−0.1	73+/−5	100	24
*UAS-unc79RNAi[108132]/+; UAS-dcr2/ +*	23.8+/−0.1	104+/−6	100	23
*UAS-unc80RNAi[108934]/+; UAS-dcr2/ +*	24.0+/−0.1	69+/−6	100	27
*timGAL4/ VIE-260B; UAS-dcr2/ +*	24.3+/−0.0	64+/−5	94	53
*timGAL4/ UAS-naRNAi[103754]; UAS-dcr2/ +*	22.5	1+/−0	2	41
*timGAL4/ UAS-unc79RNAi[108132]; UAS-dcr2/ +*	NA	0+/−0	0	35
*timGAL4/ UAS-unc80RNAi[108934]; UAS-dcr2/ +*	NA	0+/−0	0	40
*pdfGAL4 UAS-dcr2/ VIE-260B*	24.4+/−0.1	70+/−7	97	30
*pdfGAL4 UAS-dcr2/ UAS-naRNAi[103754]*	23.1+/−0.5	5+/−2	19	31
*pdfGAL4 UAS-dcr2/ UAS-unc79RNAi[108132]*	24.3+/−0.7	8+/−2	38	32
*pdfGAL4 UAS-dcr2/ UAS-unc80RNAi[108934]*	24.5+/−1.0	4+/−1	13	31

### Drosophila na, unc79, and unc80, exhibit an interdependent, post-transcriptional regulatory relationship

**Figure 3:**
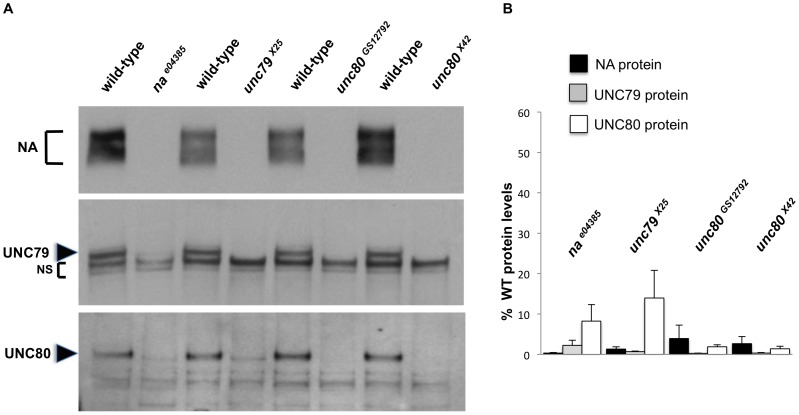
*Drosophila* NA, UNC79, and UNC80 exhibit an interdependent regulatory relationship. (A) Representative Western blot analyses, performed from adult *Drosophila* head extracts. The strains assayed were generated by backcrossing *na^e04385^* (Lanes 1–2), *unc79^x25^* (Lanes 3–4), *unc80^x42^* (Lanes 5–6), or *unc80^GS12792^* (Lanes 7–8) to *iso31* for 6–8 generations. NS  =  non-specific UNC79 bands; see *Materials and Methods* for more details. (B) Quantitation of NA, UNC79, and UNC80 protein levels in each mutant strain, as a percentage of the level observed in the corresponding wild-type strain. Black bars indicate NA protein, gray bars UNC79 protein, and white bars UNC80 protein. Error bars indicate standard error of the mean, determined from 3 independent experiments. NA, UNC79, and UNC80 protein levels are significantly lower in each mutant strain compared to the corresponding control strain, as determined by Student’s t-test (all P<0.01, except UNC80 levels in *na^e04358^*, P  =  0.012).

To examine the regulatory relationship between NA and the putative channel subunits UNC79 and UNC80, we assessed protein and transcript levels in head extracts obtained from *na, unc79,* and *unc80* mutants. It has previously been shown that *unc79^f03453^* mutants express decreased levels of NA protein, but normal levels of *na* transcript [7]. Consistent with this finding, we observe that *unc79^x25^* mutants express little or no detectable NA protein (**Figure 3A**, top panel, lanes 3–4; **Figure 3B**, black bars, P<0.01 compared to *unc79*+ control), but only a minor decrease in *na* transcript (**Figure S3A**, black bars, P<0.05). We also find that *unc79^x25^* flies express strongly decreased levels of UNC80 protein (**Figure 3A** bottom panel; **Figure 3B**, white bars, P<0.01), but no significant change in *unc80* transcript (**Figure S3A**, white bars). These data support a post-transcriptional regulatory relationship among the putative subunits. Similarly, we find that *unc80^x42^* and *unc80^GS12792^* mutants express very little NA or UNC79 protein relative to wild-type control strains (**Figure 3A**, lanes 5-8; **Figure 3B**, black and gray bars, P<0.01), while *unc80^x42^* mutants express normal levels of *na* and *unc79* transcript (**Figure S3B**). In addition, we assessed the dependence of UNC79 and UNC80 on *na*. UNC79 and UNC80 protein levels are strongly decreased in *na^e04385^* mutants (**Figure 3A**, lanes 1–2; **Figure 3B**, gray and white bars, P<0.05). While *unc79* and *unc80* transcript levels are also somewhat lower in *na^e04385^* mutants than controls (**Figure S3C**, P<0.05), the observed decreases in transcript levels (∼23%) appear insufficient to explain the decreases in protein expression (> =  86%). Notably, pan-neuronal RNAi knockdown of *na, unc79,* or *unc80* is associated with reduced expression of the other two proteins (**Figure S2B**). Taken together, these data indicate that the expression of NA, UNC79, and UNC80 proteins is strongly interdependent.

### Expression of full-length or truncated UNC79 or UNC80 restores rhythmicity and channel subunit expression to the corresponding mutant strain

**Figure 4:**
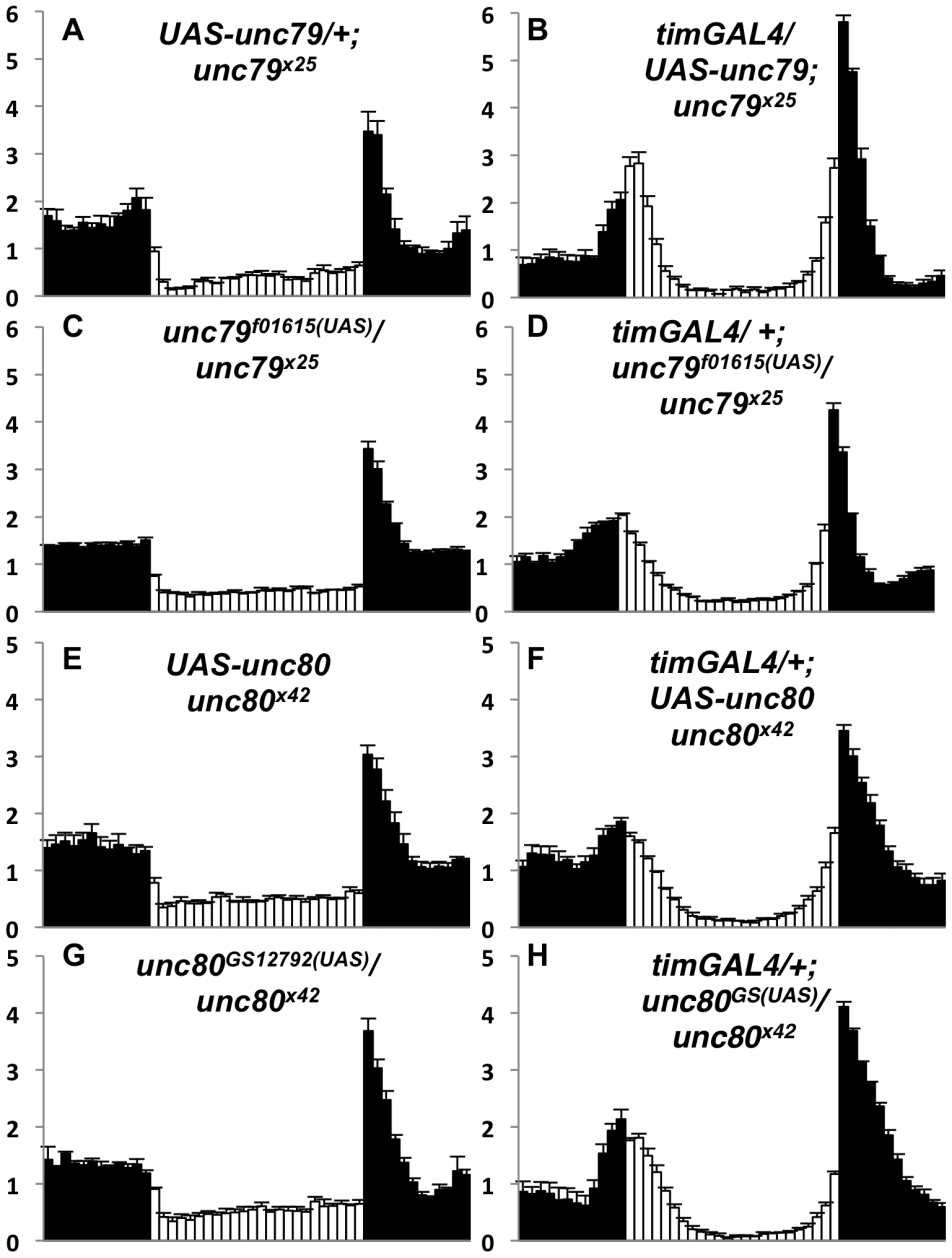
Transgenic rescue of *unc79* and *unc80* anticipation phenotypes. (A-H) Normalized activity profiles from adult males averaged over four days of LD conditions. White bars indicate light phase; black bars indicate dark phase. Error bars represent standard error of the mean. (A) *UAS-unc79MYC 23-24/+; unc79^x25^* (n = 42), MAI  =  1.1+/0.2, EAI  =  0.5+/−0.1; (B) *timGAL4*/ *UAS-unc79MYC 23-24; unc79^x25^* (n = 68), MAI  =  1.7+/−0.1, EAI  =  2.6+/−0.2; (C) *unc79^f01615^/ unc79^x25^* (n = 117), MAI  =  0.4+/−0.1, EAI  =  0.26+/−0.03; (D) *timGAL4*/ *+; unc79^f01615^/ unc79^x25^* (n = 74), MAI  =  1.1+/−0.1, EAI  =  1.6+/−0.1; (E) *UAS-HAunc80 1M unc80^x42^/ unc80^x42^* (n = 52), MAI  =  0.4+/−0.1, EAI  =  0.3+/−0.1; (F) *timGAL4*/ +; *UAS-HAunc80 1M unc80^x42^/ unc80^x42^* (n = 48), MAI  =  1.1+/−0.2, EAI  =  1.5+/−0.1; (G) *unc80^GS12792 (UAS)^/ unc80^x42^* (n = 71), MAI  =  0.5+/−0.1, EAI  =  0.3+/−0.1; (H) *timGAL4*/ *+; unc80^GS12792 (UAS)^/ unc80^x42^* (n = 43), MAI  =  1.7+/−0.1, EAI  =  1.1+/−0.1. MAI and EAI values differ significantly between each mutant (A, C, E, G) and the corresponding rescue genotype (B, D, F, H), as determined by Kruskal-Wallis one-way ANOVA (all comparisons P<0.001, 1 degree of freedom).

**Figure 5:**
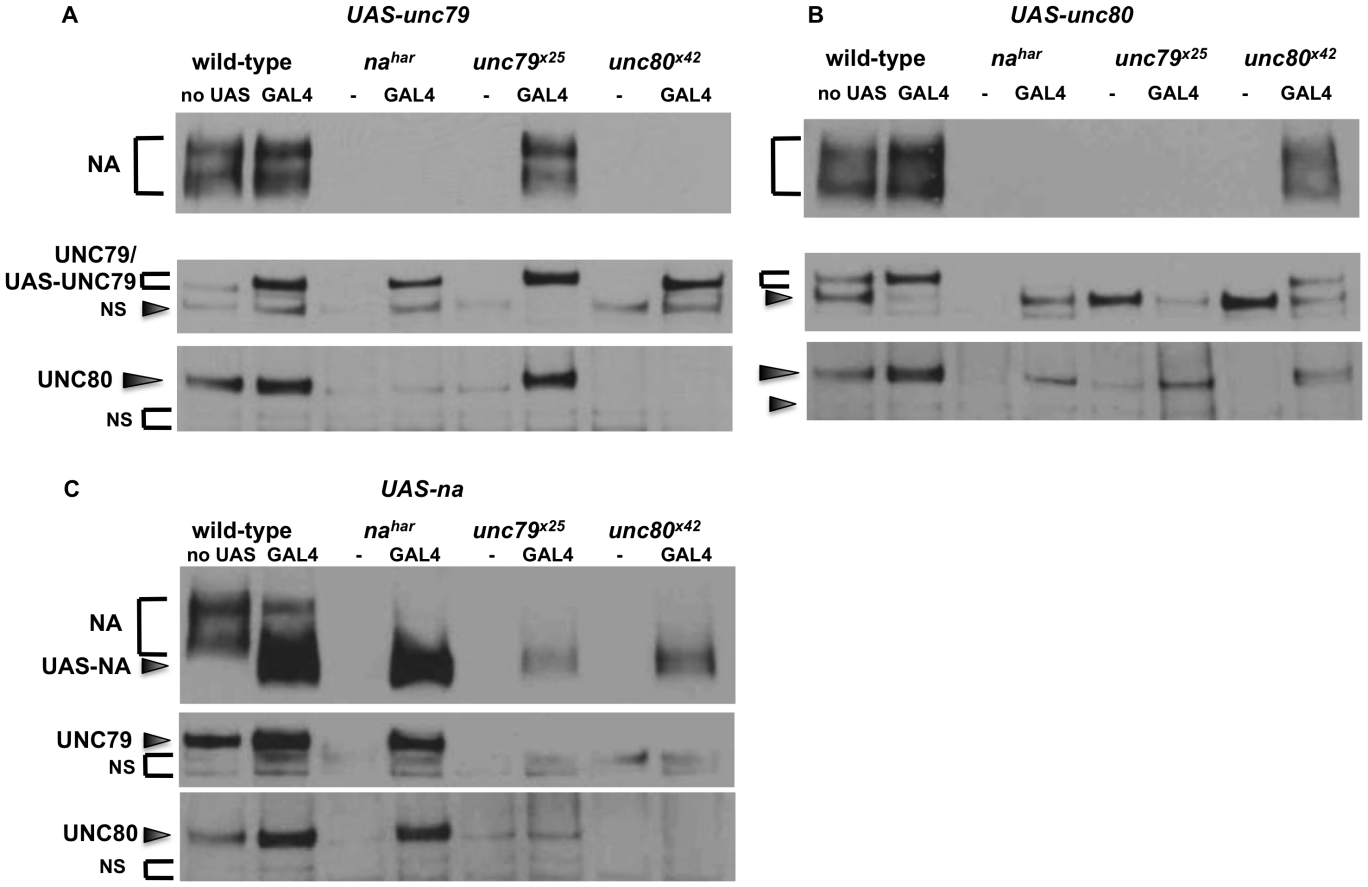
Transgenic *unc79, unc80* or *na* expression produces increased protein levels in wild-type and mutant backgrounds. (A-C) Western blot analyses were used to label UNC79, UNC80, and NA proteins from adult head extracts; representative blots are shown. For all blots shown, lane 1  =  *elav*GAL4/+ (no UAS); lanes 3,5,7  =  *UAS/+* (no GAL4); lanes 2,4,6,8  =  *elavGAL4; UAS/+.* (A) Pan-neuronal expression of *UAS-unc79MYC 23-24* in wild-type (lane 2), *na^har^* (lane 4), *unc79^x25^* (lane 6), or *unc80^x42^* (lane 8) backgrounds. (B) Expression of *UAS-HAunc80 1M* in wild-type (lane 2), *na^har^* (lane 4), *unc79^x25^* (lane 6), or *unc80^x42^* (lane 8) backgrounds. (C) Expression of *UAS-na U3* in wild-type (lane 2), *na^har^* (lane 4), *unc79^x25^* (lane 6), or *unc80^x42^* (lane 8) backgrounds.

To further assess the functional requirements for *unc79* and *unc80,* we performed tissue-specific transgenic expression experiments. Expression of full-length *unc79* cDNA in pacemaker neurons using *timGAL4* strongly restores circadian behavior in *unc79^x25^* mutants in both LD and DD conditions (**Figure 4A, B**, P<0.05; **Table 3**, P<0.001). Moreover, an intronic insertion in *unc79* that contains a UAS element (*unc79^f01615^)* also rescues behavior in the presence of *timGAL4* (**Figure 4C, D**, P<0.05; **Table 3**, P<0.001). Based on the position of this insertion, we predict that this UAS element initiates the production of functional, truncated form(s) of UNC79 (**Figure S1A**). Indeed, at least two truncated UNC79 protein bands can be detected in *GAL4/ unc79^f01615^* flies via Western blot that are not evident in wild-type flies (data not shown). These data suggest that UNC79 function in pacemaker neurons is sufficient to promote rhythmicity. We also assessed whether transgenic expression of UNC79 restores NA and UNC80 expression in *unc79* mutants. For these assays we utilized *elavGAL4*, as this pan-neuronal driver is likely more representative of endogenous channel complex expression than *timGAL4* [3]. As described earlier, *elavGAL4* driven expression of RNAi to *na, unc79,* or *unc80* leads to strong knockdown of the corresponding proteins in adult head extracts, indicating that this driver encompasses the major sources of expression of all three gene products in the *Drosophila* head (**Figure S2A**). We find that pan-neuronal expression of either full-length or truncated UNC79 strongly restores NA and UNC80 protein levels to *unc79* mutants (**Figure 5A** **,** lanes 5-6, P<0.01; data not shown).

**Table 3:** Transgenic rescue of *unc79* and *unc80* rhythmicity phenotypes.

Genotype	Period (hrs)	Power	Rhythmic (%)	n
*timGAL4/ +*	24.6+/−0.0	40+/−3	76	135
*timGAL4/ UAS-na*	25.3+/−0.1	21+/−6	55	22
*timGAL4/ UAS-unc79MYC*	25.0+/−0.1	59+/−7	88	33
*timGAL4/ +; unc79[UAS-f01615]/ +*	25.0+/−0.1	85+/−7	92	36
*timGAL4/ +; UAS-HAunc80/ +*	25.0+/−0.2	38+/−7	69	35
*timGAL4/ +; unc80[UAS-GS12792]/ +*	24.6+/−0.1	83+/−9	95	19
*timGAL4/ +; unc79[x25]*	25.7+/−3.1	2+/−1	7	56
*UAS-unc79MYC/+; unc79[x25]*	24.1+/−2.4	3+/−1	13	30
*unc79[x25]/ unc79[UAS-f01615]*	24.6+/−0.3	2+/−1	6	100
*timGAL4/ UAS-unc79MYC; unc79[x25]*	23.8+/−0.0	108+/−6	97	61
*timGAL4/ +; unc79[x25]/ unc79[UAS-f01615]*	24.4+/−0.1	58+/−5	82	66
*timGAL4/ UAS-na; unc79[x25]* 1	N/A	1+/−1	0	2
*timGAL4/ +; UAS-HAunc80 unc79[x25]/ unc79[x25]*	21.8+/−5.8	1+/−0	3	75
*timGAL4/ +; unc79[x25] unc80[UAS-GS12792]/ unc79[x25]*	22.4+/−1.6	3+/−1	12	50
*timGAL4/ UAS-na; UAS-HAunc80 unc79[x25]/ unc79[x25]*	24.0	1+/−0	4	28
*timGAL4/ +; unc80[x42]*	25.0+/−1.0	1+/−0	3	66
*unc80[x42] UAS-HAunc80/ unc80[x42]*	19.5+/−0.0	1+/−0	3	66
*unc80[x42]/ unc80[UAS-GS12792]*	N/A	0+/−0	0	54
*timGAL4/ +; UAS-HAunc80 unc80[x42]/ unc80[x42]*	24.5+/−0.1	94+/−7	93	42
*timGAL4/ +; unc80[x42]/ unc80[UAS-GS12792]*	24.3+/−0.1	134+/−5	100	38
*timGAL4/ UAS-na; unc80[x42]* 1	N/A	2+/−2	0	5
*timGAL4/ UAS-unc79MYC; unc80[x42]*	17.8+/−1.5	2+/−0	5	61
*timGAL4/ +; unc79[UAS- f01615] unc80[x42]/* *unc80[x42]*	19.5	1+/−0	2	48

**Figure 6:**
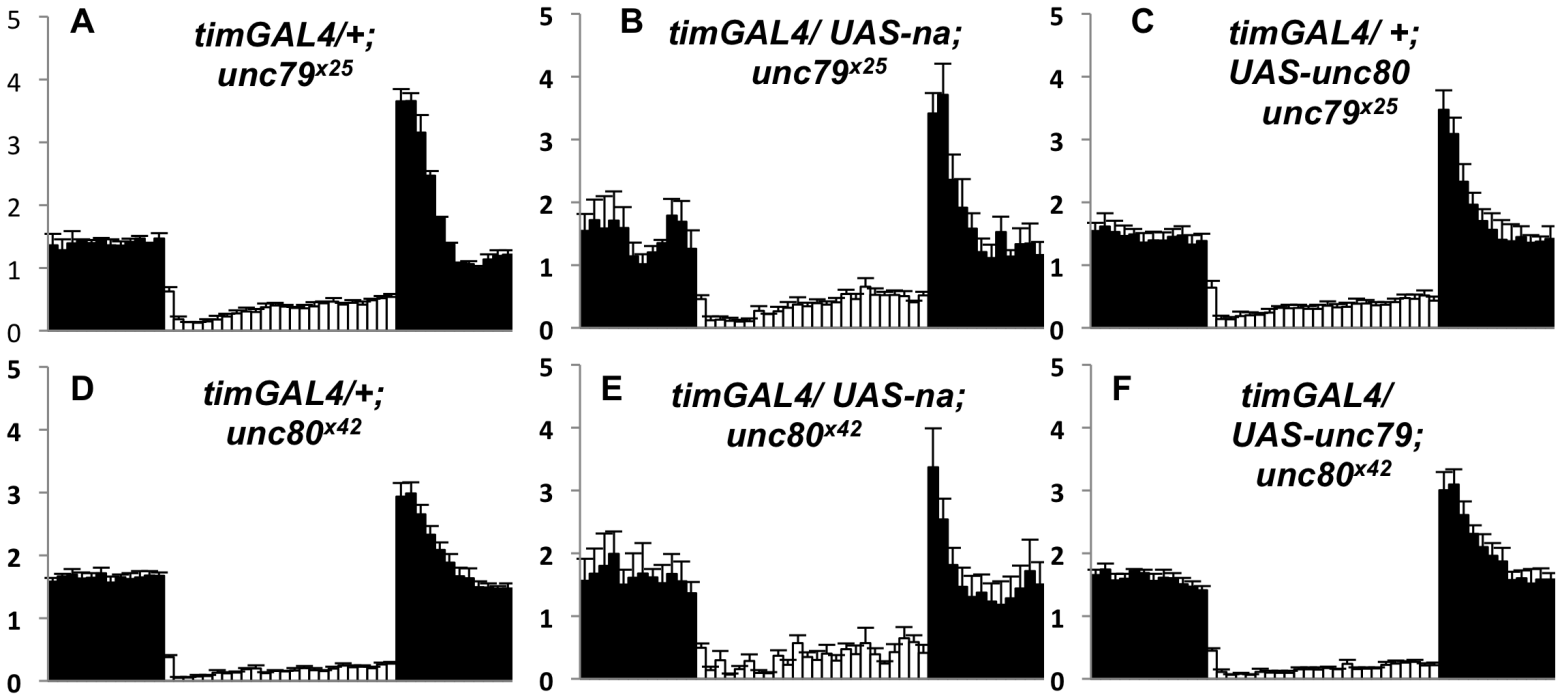
Transgenic expression of other subunits does not restore anticipatory behavior to *unc79* or *unc80* mutants . (A-F) Normalized activity profiles from adult males averaged over four days of LD conditions. White bars indicate light phase; black bars indicate dark phase. Error bars represent standard error of the mean. (A) *timGAL4*/ +; *unc79^x25^* (n = 73), MAI  =  0.6+/−0.1, EAI  =  0.3+/−0.1; (B) *timGAL4*/ *UAS-na U3; unc79^x25^* (n = 25), MAI  =  1.2+/−0.2, EAI  =  0.3+/−0.1; (C) *timGAL4*/+; *UAS-HAunc80 1M unc79^x25^*/ *unc79^x25^* (n = 90), MAI  =  0.4+/−0.1, EAI  =  0.32+/−0.04; (D) *timGAL4*/ *+*; *unc80^x42^* (n =  78), MAI  =  0.3+/−0.1, EAI  =  0.16+/−0.03; (E) *timGAL4*/ *UAS-na U3; unc80^x42^* (n = 21), MAI  =  0.6+/−0.2, EAI  =  0.5+/−0.2; (F) *timGAL4*/ *UAS-unc79MYC 23-24*; *unc80^x42^* (n = 74), MAI  =  0.4+/−0.1, EAI  =  0.19+/−0.04. For *unc79^x25^* genotypes (A-C), EAI values do not differ significantly (Kruskal-Wallis one-way ANOVA, 2 degrees of freedom, P  =  0.567). MAI values for genotypes A-C exhibit significant variation (Kruskal-Wallis one-way ANOVA, 2 degrees of freedom, P  =  0.0006), but neither of the transgenic genotypes (B,C) differs significantly from the mutant control (A; Dunn’s method). For *unc80^x42^* genotypes (D-F), no significant difference is detected in MAI values (Kruskal-Wallis one-way ANOVA, 2 degrees of freedom, P  =  0.391). Significant variation is observed for EAI values for genotypes D-F (Kruskal-Wallis one-way ANOVA, 2 degrees of freedom, P  =  0.022), but no pairwise differences are significant (Dunn’s method).

1 Few flies survived to the end of DD; refer to Figures 6B,E for LD phenotype.

Similarly, expression of full-length *unc80* cDNA using *timGAL4* strongly rescues LD and DD rhythmicity in *unc80^x42^* mutant flies (**Figure 4E, F**, P<0.05; **Table 3**, P<0.001). Surprisingly, we also find that *timGAL4* restores LD and DD rhythmic behavior in *unc80^GS12792^/ unc80^x42^* trans-heterozygotes (**Figure 4G, H**; P<0.05; **Table 3**, P< 0.001). As described, *unc80^GS12792^* contains a UAS element near the middle of the presumptive UNC80 coding sequence (**Figure S1B**). We predict that this UAS element is capable of initiating the production of functional UNC80 protein, which likely contains < =  1790 of the ∼3310 amino acids predicted for the full length protein. Consistent with this prediction, we detect at least one truncated (∼180 Kd) UNC80 band in *GAL4/ unc80^GS12792^* flies (data not shown). These data indicate that broad circadian expression of either full-length or C-terminal UNC80 protein is sufficient to restore rhythmicity to *unc80* mutants. In addition, we find that pan-neuronal expression of either full-length or truncated UNC80 restores NA and UNC79 protein expression to *unc80* mutants (**Figure 5B**, lanes 7–8, P<0.01; data not shown).

### Transgenic expression of NA, UNC79, or UNC80 does not relieve the requirement for other channel subunits in the Drosophila brain

Given the interdependent regulatory relationship among *na, unc79,* and *unc80*, we asked whether transgenic expression of any putative subunits could restore rhythmicity in the absence of another. Notably, transfection experiments in mouse primary brain culture indicate that expression of mouse UNC80 can bypass the requirement for UNC79 [11]. In contrast, we find that circadian or pan-neuronal expression of either NA or UNC80 *in vivo* is not sufficient to restore rhythmicity to *unc79^x25^* mutants (**Figure 6A-C**; **Table 3**, P  =  0.401; data not shown), nor is co-expression of both NA and UNC80 (**Table 3** **,** P  =  0.661; data not shown). Similarly, circadian or pan-neuronal expression of either NA or UNC79 does not restore rhythmicity to *unc80^x42^* mutants (**Figure 6D-F**; **Table 3**, P > =  0.671; data not shown). Because each mutant is deficient in the expression of all three proteins, we also assessed whether pan-neuronal expression of one subunit restores protein expression in another mutant background. We find that transgenic UAS-*na* cDNA expression in *unc79* or *unc80* mutants produces a detectable increase in NA protein levels (**Figure 5C** **,** top panel; **Figure S4C**, P<0.01), but much less than what is observed when expressed in a *na* mutant background (**Figure 5C** **,** top panel; **Figure S4C**, P<0.05). This indicates that transgenic NA expression is strongly, but not completely, dependent on *unc79* and *unc80*. Transgenic NA expression in *unc79^x25^* mutants results in a small but significant increase in UNC80 protein levels (**Figure 5C** **,** bottom panel; **Figure S4C,** right panel, P<0.05), suggesting that NA/UNC80 regulation may occur independently of UNC79. However, transgenic NA does not restore a significant increase in UNC79 to *unc80^x42^* mutants (**Figure 5C** **,** middle panel; **Figure S4C,** right panel, P  =  0.12). Expression of UAS-*HAunc80* cDNA results in increased levels of UNC80 protein in *na^har^* and *unc79^x25^* mutants (**Figure 5B** **,** bottom panel; **Figure S4B**, P<0.01). While transgenic *unc80* produces no clear increase in NA levels in *unc79^x25^* mutants (P  =  0.65), it does produce a significant increase in UNC79 levels in *na^har^* mutants (**Figure 5B** **,** top and middle panels; **Figure S4B,** right panel; P<0.01). This suggests that regulation of UNC79 expression by UNC80 can occur independently of NA. Finally, transgenic expression of UAS-*unc79* also produces a clear increase in UNC79 protein expression in both *na* and *unc80* mutants (**Figure 5A** **,** middle panel; **Figure S4A**, P<0.01). However, *unc79* expression does not result in increased UNC80 expression in *na* mutants, nor does it restore NA expression to *unc80* mutants (**Figure 5A** **,** top and bottom panels; **Figure S4A,** right panel, P > =  0.25). Taken together, these data indicate that increasing expression of one or two putative channel subunit proteins in the absence of a third does not improve behavioral rhythmicity.

### NA, UNC79, and UNC80 proteins are present in a complex in the Drosophila head

**Figure 7:**
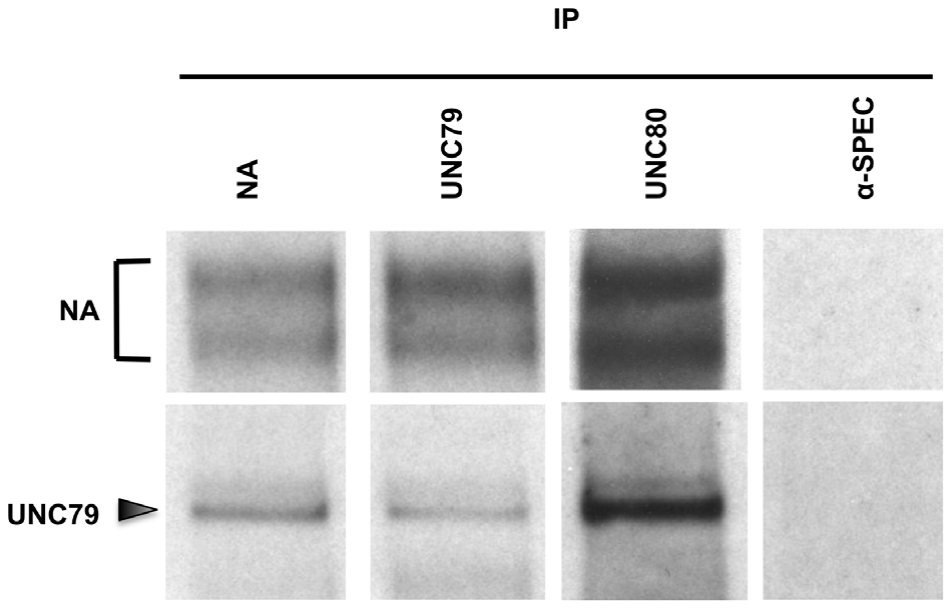
NA, UNC79, and UNC80 proteins form a complex in the *Drosophila* head. Western blot analyses of immunoprecipitated complexes. Immunoprecipitations were performed from membrane preparations of adult *Drosophila* head extracts using the antibodies indicated (anti-NA, anti-UNC79, anti-UNC80, and anti-α-SPECTRIN; see *Materials and Methods*).

The mammalian orthologs of NA, UNC79, and UNC80 are believed to function in a protein complex [11], [13]. To determine whether the *Drosophila* proteins also interact with each other, we performed co-immunoprecipitation experiments from adult head extracts. We find that NA protein can be immunoprecipitated using anti-UNC79 or anti-UNC80, while UNC79 protein is immunoprecipitated using either anti-NA or anti-UNC80 (**Figure 7**). These data support a model in which *Drosophila* NA, UNC79, and UNC80 proteins form a channel complex, similar to the proposed relationship of their mammalian orthologs.

## Discussion

Here we demonstrate that the *Drosophila* genes *unc79* and *unc80 (aka CG18437)* are required for robust circadian locomotor rhythms. We find that a previously described *unc79* mutant allele, *unc79^f03453^*, exhibits circadian phenotypes that are less severe than strong loss-of-function *na* mutants (**Figure 1** **,** **Table 1**). It was initially unclear whether the differences in phenotypic severity represented partial *unc79* gene function in the *unc79^f03453^* mutant or residual NA channel function in the absence of *unc79.* Notably, *UNC79* knockout mice retain NALCN expression and some basal channel activity [11]. For *unc79^f03453^*, transcript analysis indicates that this allele is likely hypomorphic. A novel deletion allele, *unc79^x25^*, represents a complete or near complete loss of *unc79* function, and these mutants exhibit severe circadian phenotypes that are indistinguishable from *na* mutants. Similarly, mutants that contain a coding disruption in the *Drosophila* UNC80 ortholog (*unc80/ CG18437)*, exhibit strong rhythmicity defects that are very similar to *na* and *unc79^x25^* mutants (**Figure 1** **,** **Table 1**).

Expression data from *na, unc79,* and *unc80* mutants indicate an interdependent, post-transcriptional regulatory relationship between these gene products in *Drosophila* (**Figure 3** **, Figure S3**). This is similar to previous findings from *C. elegans,* perhaps reflecting conserved regulatory mechanisms [7], [10]. Notably, residual UNC79 and UNC80 protein is observed in *na^e04385^* mutants and some UNC80 protein is detected in *unc79^x25^* mutants. The *na^e04385^* allele contains a transposon insertion in the middle of the NA coding sequence, and behavioral analyses suggest that this represents a severe loss of function allele (**Figure 1** **,** **Table 1**; [7]). We have no evidence of phenotypic differences among the three mutants that might correspond to the residual protein expression. Strong *na, unc79,* and *unc80* alleles in the *iso31* background exhibit a similar array of phenotypes in addition to circadian behavioral defects, including decreased locomotor activity, poor female fertility, and poor viability in locomotor assays (data not shown). Different isogenized strains retain some variability in the severity of these phenotypes, but this does not appear to correlate with mutation of a particular gene (data not shown). The regulatory relationship among these gene products in *Drosophila* appears to be somewhat different than in mammals. In mice, *UNC79* knockouts lack UNC80 expression but retain NALCN. While both *NALCN* and *UNC79* knockouts exhibit breathing rhythm defects and die shortly after birth, slight differences in neonatal lethality between the two mutants may reflect residual NALCN activity in the *UNC79* knockout [11].

Our data support the hypothesis that UNC79 and UNC80 function in a complex with NA in circadian pacemaker neurons to promote rhythmic behavior. The anatomical requirements for the channel complex includes the PDF-expressing (PDF+) subset of pacemaker neurons, since *pdf*GAL4 driven RNAi knockdown of *na, unc79,* or *unc80* results in decreased DD rhythmicity (**Table 2**). It is notable that *pdf*GAL4 driven knockdown of *na, unc79,* or *unc80* does not cause strong defects in LD anticipatory behavior (**Figure 2**). This phenotype is less severe than what is observed in *pdf* neuropeptide mutants [20], suggesting that PDF release is not completely compromised upon loss of NA channel complex function. These findings complement previous *na* tissue-specific rescue data, in which expression of the GAL4 inhibitor GAL80 in PDF+ neurons (*pdfGAL80*) blocks *elavGAL4* and *timGAL4* mediated rescue of DD rhythmicity but does not block LD rescue [4].

Transgenic rescue experiments indicate that either full length or C-terminal regions of UNC79 and UNC80 fully restore behavioral rhythmicity to the corresponding mutant strains (**Figure 4** **,** **Table 3**). For both *unc79* and *unc80,* a UAS insertion after the presumed start methionine can initiate the production of a functional, truncated protein. The robust rescue observed using the *unc80^GS12792-UAS^* strain was particularly surprising, as conceptual translation indicates that the protein produced would include, at most, ∼1790 of the ∼3310 amino acids predicted for full-length *Drosophila* UNC80 (**Figure S1B**). Notably, this C-terminal region exhibits greater homology to mammalian UNC80 than does the N-terminal region preceding the *GS12792* insertion (data not shown). These rescue data imply that the C-terminal ∼half of UNC80 protein may be sufficient to promote circadian behavior and NA/UNC79 protein expression. However, we cannot rule out cooperative function with residual N-terminal UNC80 protein that may be present in *unc80^x42^* and *unc80^GS12792^* mutants. Similarly, the C-terminal truncated protein produced in GAL4/ *unc79^f01615^* flies (containing < = 1871 of the ∼2776 aa) could potentially cooperate with residual N-terminal UNC79 protein in *unc79^x25^* and *unc79^f01615^* mutants.

We also used transgenic expression experiments to address whether both *unc79* and *unc80* are required for more than regulating subunit protein levels. In mice, UNC80 has been implicated in the regulation of NALCN channel activity, whereas the requirement for UNC79 in cultured hippocampal neurons can be bypassed by UNC80 transfection [11], [13]. In contrast, we find that the *in vivo* requirement for one subunit in the *Drosophila* circadian system cannot be bypassed by expression of another. Transgenic expression of *Drosophila na* and/or *unc80* promotes significant expression of the corresponding protein(s) to *unc79^x25^* mutants but provides no measureable improvement in rhythmic behavior (**Figure 5** **,** **Figure 6** **,** **Table 3**, data not shown). In contrast, some hypomorphic alleles (*unc79^f03453^)* and RNAi combinations have been identified in which little subunit protein is detected but significant rhythmicity is observed (**Figure 1** **,** **Table 1**, data not shown; [7]). Taken together, these data suggest that *Drosophila* UNC79 and UNC80 each have functional requirements beyond promoting the expression of other subunits. These putative auxiliary subunits could be required for proper localization of the channel complex and/or regulation of channel activity. As UNC80 is known to mediate NALCN activity regulation in mammals, it will be valuable to determine whether similar or distinct mechanisms modulate channel activity in circadian pacemaker neurons. Such regulatory mechanisms may be relevant to the mammalian circadian pacemaker, the suprachiasmatic nucleus (SCN), as electrophysiological analyses suggest that NALCN may indeed function in this tissue [26], [27], [28].

Finally, we note that transgenic expression of UNC80 in *na* mutants produces a significant increase in UNC79 expression, and transgenic NA expression in *unc79* mutants produces a small but significant increase in UNC80 expression (**Figure 5** **, Figure S4**). If the regulatory relationship among these gene products is mediated by protein-protein interaction, this would suggest that UNC79 and UNC80 can interact in the absence of NA, and that NA and UNC80 can interact in the absence of UNC79. This model for channel subunit interactions is supported by co-immunoprecipitation data in mammalian cell culture, where UNC80 was found to mediate the physical interaction between NALCN and UNC79 [11].

## Supporting Information

Figure S1 ***Drosophila unc79*** **and** ***unc80*** **gene loci.** Schematic representation of the *Drosophila unc79* (A) and *unc80* (B) gene loci, both located on Chromosome 3R. Transcript and protein predictions are based on *Drosophila* genome annotation 5.1. Triangles represent the approximate locations of relevant transposable elements insertions. P  =  P-element transposon insertion; PBac  =  Piggybac transposon insertion. Black triangles represent insertions that were evaluated as mutant alleles and/or were used to generate novel alleles. Gray triangles represent insertions that contain a 5’- 3’ UAS element; both of these insertions likely decrease gene function in the absence of GAL4 but produce functional proteins in the presence of GAL4. (A) *unc79* gene locus. Bracket indicates the genomic sequence deleted in the *unc79^x25^* allele. This includes 57 bp and 80 bp coding exons. M =  approximate location of the first 3 predicted start methionines after the *unc79 ^f01615-UAS^* insertion. (B) *unc80/ CG18437* gene locus. M(4) =  exon containing the first 4 predicted start methionines after the *unc80^GS12792-UAS^* insertion.
(TIF)

Figure S2 **Pan-neuronal expression of** ***na*** **,** ***unc79*** **, or** ***unc80*** **RNAi decreases expression of channel complex proteins.** Quantitation of NA (black bars), UNC79 (gray bars), and UNC80 (white bars) protein levels upon pan-neuronal (*elavGAL4*) driven expression of RNAi, relative to control strains (*elavGAL4; attp VIE260/+* with or without UAS-*dcr2*). Protein levels were measured from Western blot data using NIH Image J gel analysis. Error bars represent standard error of the mean (n =  4 experiments). Statistical significance was determined using Student’s t-test. n.s. =  no significant difference; *  =  P<0.05; **  =  P<0.01. (A) Decreased levels of the targeted protein are detected upon pan-neuronal expression of *na* RNAi (VDRC *103754*), *unc79* RNAi (VDRC *108132*), or *unc80* RNAi (VDRC *108934*). For *unc79* and *unc80*, knockdown of protein levels is significantly enhanced upon co-expression of UAS*-dicer2 (dcr2).* (B) Pan-neuronal RNAi knockdown of *na, unc79,* or *unc80* results in decreased expression of the other putative subunit proteins.
(TIF)

Figure S3 ***Drosophila na*** **,** ***unc79*** **, and** ***unc80*** **mutants display minimal differences in transcript expression of other subunits.** mRNA expression levels of *na* (black bars), *unc79* (gray bars), and/or *unc80* (white bars) in (A) *unc79^x25^* mutants, (B) *unc80^x42^* mutants, and (C) *na^e04385^* mutants relative to expression in the corresponding wild-type strains, as determined by qPCR. The strains assayed were backcrossed to *w^1118^ iso31* for 6–8 generations. Samples were normalized to RP49 expression and analysed using theΔΔCt method, as described in *Materials and Methods*. Error bars represent standard error of the mean. Statistical significance was determined using Student’s t-test. n.s. =  no significant difference; *  =  P<0.05; **  =  P<0.01.
(TIF)

Figure S4 **Quantitation of protein levels upon transgenic** ***unc79*** **,** ***unc80*** **or** ***na*** **expression.** Quantitation of NA (black bars), UNC79 (gray bars), and UNC80 (white bars) protein levels in the genotypes indicated, as determined using NIH ImageJ analyses of Western blot data (n = 3–5 experiments). Error bars indicate standard error of the mean. In left panels, protein levels are reported as a percent of wild-type (*elavGAL4/ +*). In right panels, the % increase in expression reflects the increase in protein levels observed in *elavGAL4* UAS/+ strains as compared to UAS/+ alone, as determined within each experiment and mutant background. Statistical significance was determined using Student’s t-test. n.s. =  no significant difference; *  =  P<0.05; **  =  P<0.01. (A) Pan-neuronal expression of UAS-*unc79MYC* (*elavGAL4; UAS-unc79MYC 23*–*24/+*) in the backgrounds indicated. In the right panel, UNC80 and NA levels were decreased upon transgenic expression of *unc79*, but these changes were not significant. (B) Pan-neuronal expression of UAS-*HAunc80* (*elavGAL4;; UAS-HA-unc80 1M/+*). (C) Pan-neuronal expression of UAS-*na* (*elavGAL4; UAS-na U3 /+*).
(TIF)

Table S1 ***unc79*** **and** ***unc80*** **complementation assays.**
(DOCX)

Table S2 **DD rhythmicity in** ***unc79*** **and** ***unc80*** **single and double heterozygotes.**
(DOCX)

**Materials and Methods S1** (DOCX)
