# Supplementary figures and images for: UNC79 and UNC80, Putative Auxiliary Subunits of the NARROW ABDOMEN Ion Channel, Are Indispensable for Robust Circadian Locomotor Rhythms in Drosophila
Source: PLoS One. 2013 Nov 5;8(11):e78147. doi: 10.1371/journal.pone.0078147 (PMC3818319; doi:10.1371/journal.pone.0078147)

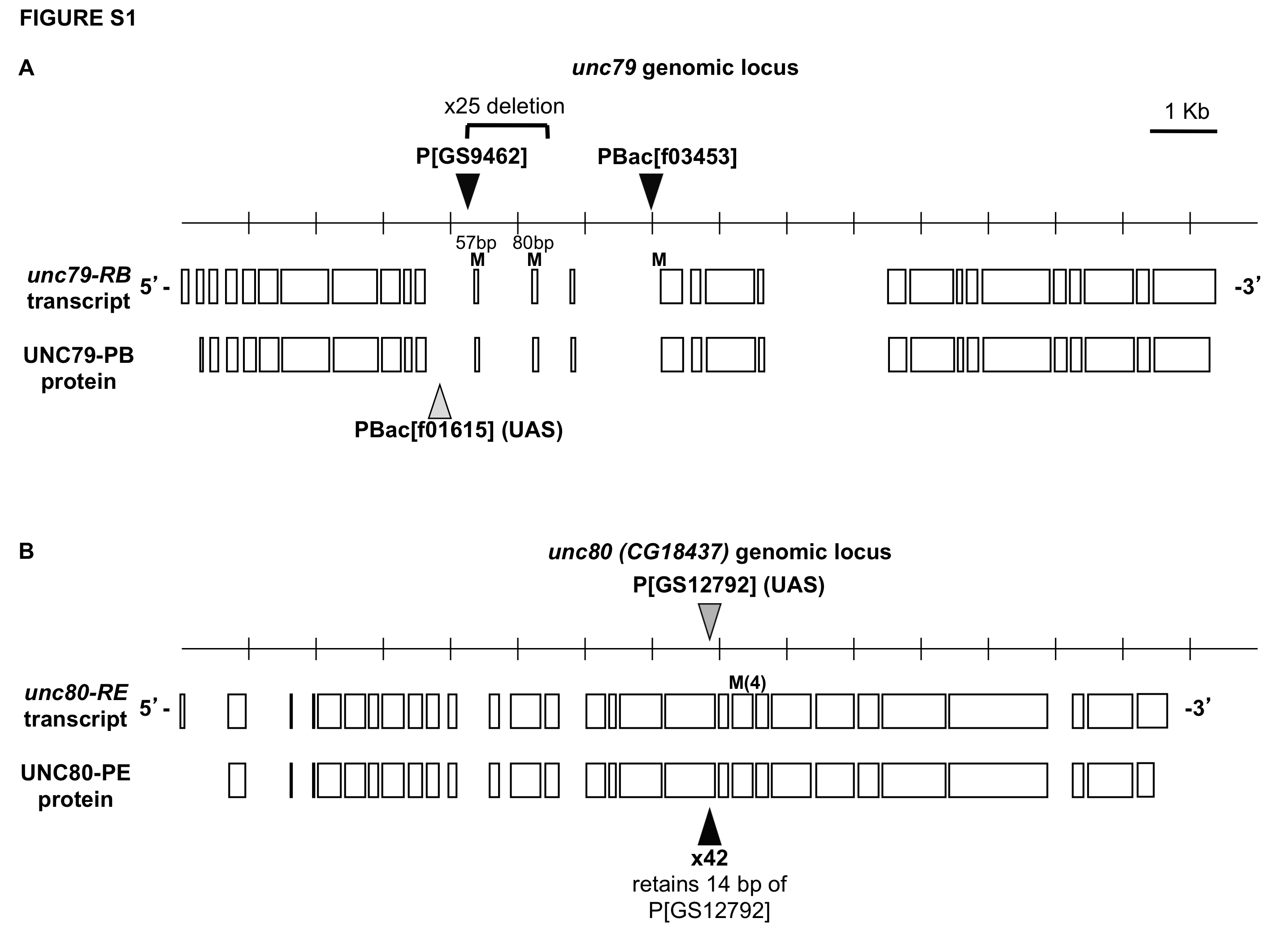

Supplement: Figure S1 — Drosophila unc79 and unc80 gene loci. Schematic representation of the Drosophila unc79 (A) and unc80 (B) gene loci, both located on Chromosome 3R. Transcript and protein predictions are based on Drosophila genome annotation 5.1. Triangles represent the approximate locations of relevant transposable elements insertions. P = P-element transposon insertion; PBac = Piggybac transposon insertion. Black triangles represent insertions that were evaluated as mutant alleles and/or were used to generate novel alleles. Gray triangles represent insertions that contain a 5’- 3’ UAS element; both of these insertions likely decrease gene function in the absence of GAL4 but produce functional proteins in the presence of GAL4. (A) unc79 gene locus. Bracket indicates the genomic sequence deleted in the unc79x25 allele. This includes 57 bp and 80 bp coding exons. M = approximate location of the first 3 predicted start methionines after the unc79 f01615-UAS insertion. (B) unc80/ CG18437 gene locus. M(4) = exon containing the first 4 predicted start methionines after the unc80GS12792-UAS insertion. (TIF) [file pone.0078147.s001.tif]

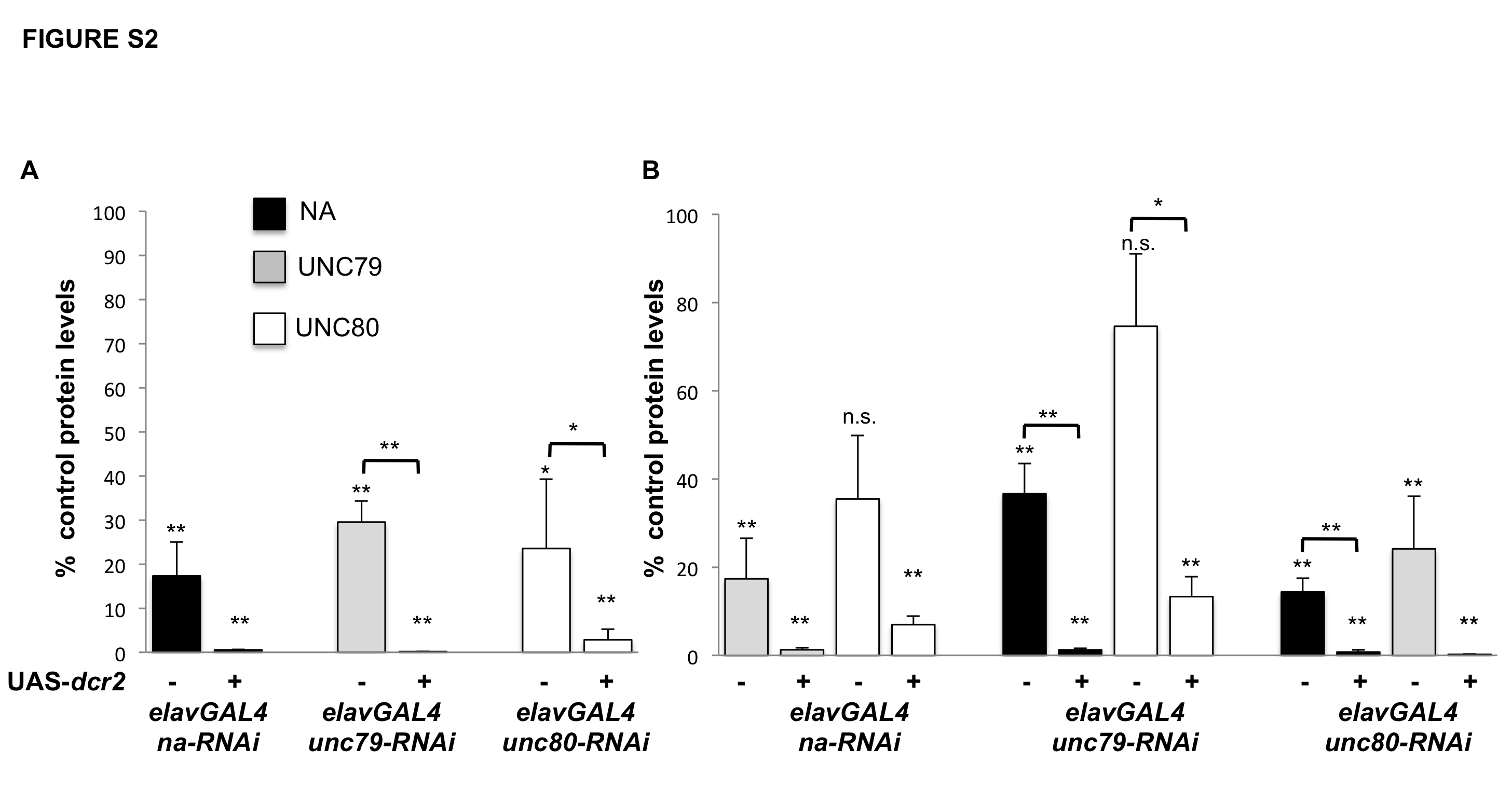

Supplement: Figure S2 — Pan-neuronal expression of na , unc79 , or unc80 RNAi decreases expression of channel complex proteins. Quantitation of NA (black bars), UNC79 (gray bars), and UNC80 (white bars) protein levels upon pan-neuronal (elavGAL4) driven expression of RNAi, relative to control strains (elavGAL4; attp VIE260/+ with or without UAS-dcr2). Protein levels were measured from Western blot data using NIH Image J gel analysis. Error bars represent standard error of the mean (n = 4 experiments). Statistical significance was determined using Student’s t-test. n.s. = no significant difference; * = P<0.05; ** = P<0.01. (A) Decreased levels of the targeted protein are detected upon pan-neuronal expression of na RNAi (VDRC 103754), unc79 RNAi (VDRC 108132), or unc80 RNAi (VDRC 108934). For unc79 and unc80, knockdown of protein levels is significantly enhanced upon co-expression of UAS-dicer2 (dcr2). (B) Pan-neuronal RNAi knockdown of na, unc79, or unc80 results in decreased expression of the other putative subunit proteins. (TIF) [file pone.0078147.s002.tif]

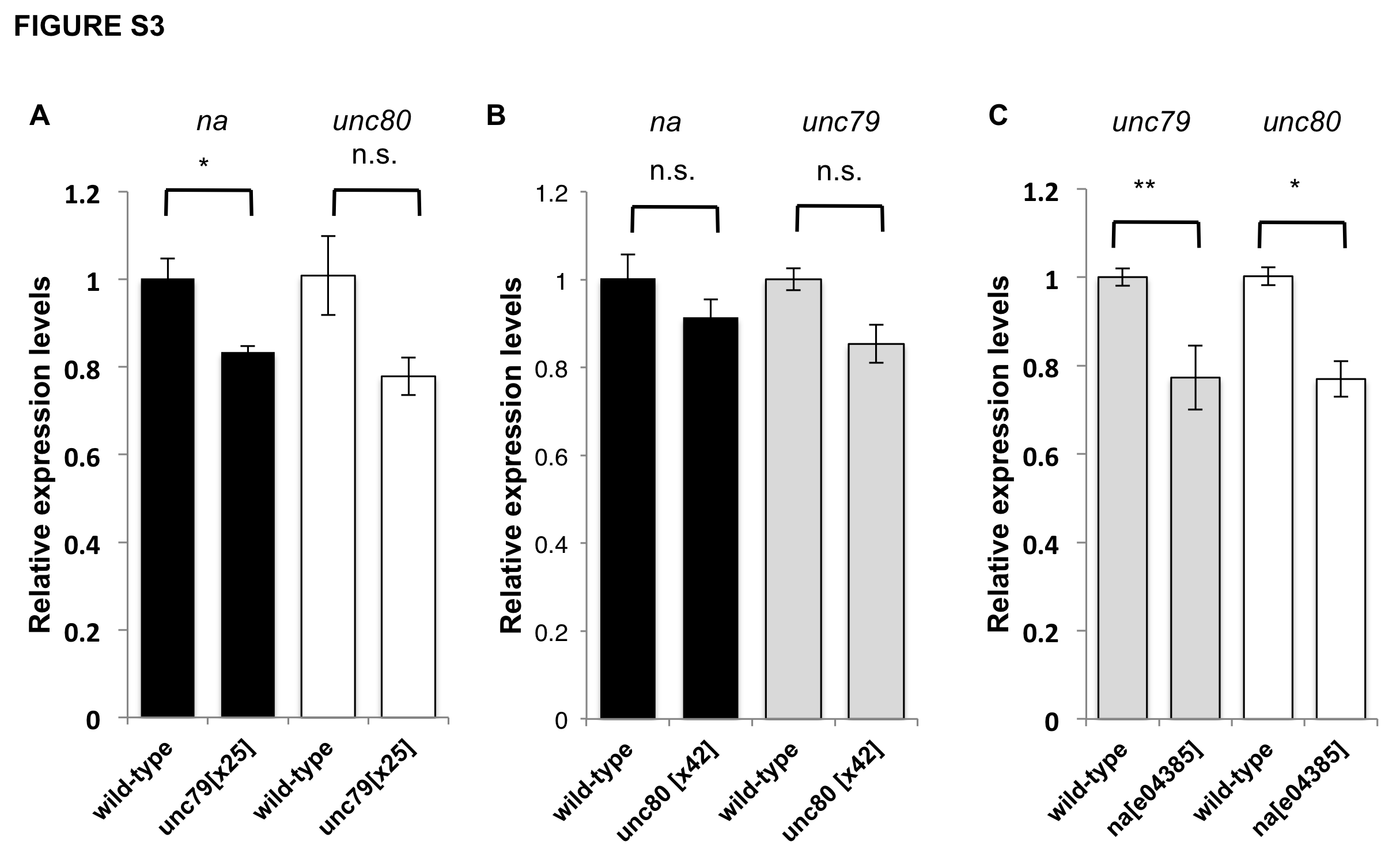

Supplement: Figure S3 — Drosophila na , unc79 , and unc80 mutants display minimal differences in transcript expression of other subunits. mRNA expression levels of na (black bars), unc79 (gray bars), and/or unc80 (white bars) in (A) unc79x25 mutants, (B) unc80x42 mutants, and (C) nae04385 mutants relative to expression in the corresponding wild-type strains, as determined by qPCR. The strains assayed were backcrossed to w1118 iso31 for 6–8 generations. Samples were normalized to RP49 expression and analysed using theΔΔCt method, as described in Materials and Methods. Error bars represent standard error of the mean. Statistical significance was determined using Student’s t-test. n.s. = no significant difference; * = P<0.05; ** = P<0.01. (TIF) [file pone.0078147.s003.tif]

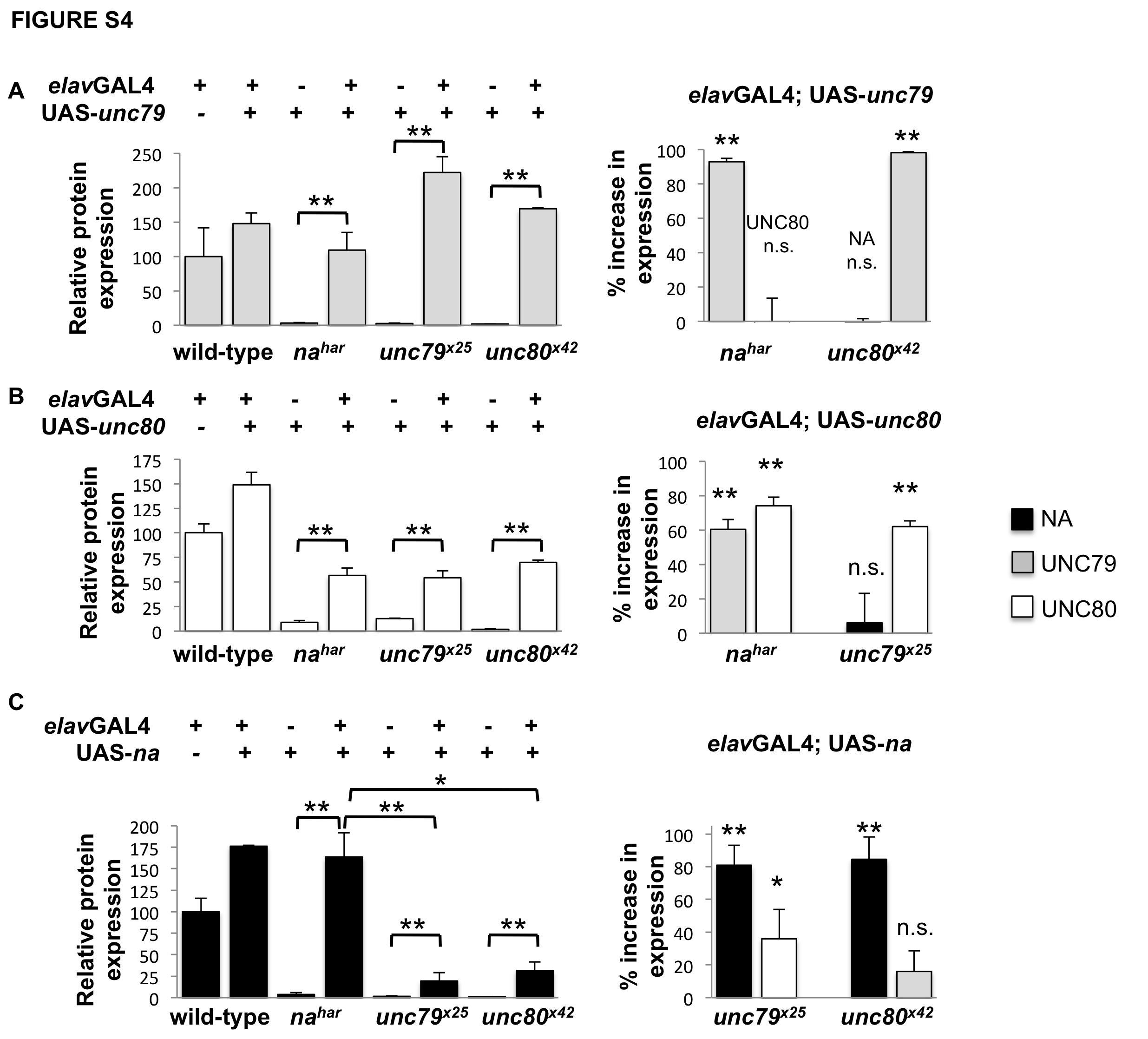

Supplement: Figure S4 — Quantitation of protein levels upon transgenic unc79 , unc80 or na expression. Quantitation of NA (black bars), UNC79 (gray bars), and UNC80 (white bars) protein levels in the genotypes indicated, as determined using NIH ImageJ analyses of Western blot data (n = 3–5 experiments). Error bars indicate standard error of the mean. In left panels, protein levels are reported as a percent of wild-type (elavGAL4/ +). In right panels, the % increase in expression reflects the increase in protein levels observed in elavGAL4 UAS/+ strains as compared to UAS/+ alone, as determined within each experiment and mutant background. Statistical significance was determined using Student’s t-test. n.s. = no significant difference; * = P<0.05; ** = P<0.01. (A) Pan-neuronal expression of UAS-unc79MYC (elavGAL4; UAS-unc79MYC 23–24/+) in the backgrounds indicated. In the right panel, UNC80 and NA levels were decreased upon transgenic expression of unc79, but these changes were not significant. (B) Pan-neuronal expression of UAS-HAunc80 (elavGAL4;; UAS-HA-unc80 1M/+). (C) Pan-neuronal expression of UAS-na (elavGAL4; UAS-na U3 /+). (TIF) [file pone.0078147.s004.tif]
