# Supplementary material for: UNC79 and UNC80, Putative Auxiliary Subunits of the NARROW ABDOMEN Ion Channel, Are Indispensable for Robust Circadian Locomotor Rhythms in Drosophila
Source: PLoS One. 2013 Nov 5;8(11):e78147. doi: 10.1371/journal.pone.0078147 (PMC3818319; doi:10.1371/journal.pone.0078147)
Supplement: Table S1 — unc79 and unc80 complementation assays. (DOCX) [file pone.0078147.s005.docx]

**TABLE S1. *unc79* and *unc80* complementation assays.**

| **Genotype ^1^** | **Period (hrs)** | **Power** | **Rhythmic (%)** | **n** |
| --- | --- | --- | --- | --- |
| +/ *Df(3R)ED5942* | 23.6 +/- 0.1 | 65 +/- 8 | 92 | 26 |
| *unc79[x25]/ Df(3R)ED5942* | NA | 0 +/- 0 | 0 | 8 |
| +/ *Df(3R)BSC499* | 23.9 +/- 0.0 | 78 +/- 5 | 91 | 69 |
| *unc80[GS12792]/ Df(3R)BSC499* | NA | 0 +/- 0 | 0 | 21 |
| *unc80[x42]/ Df(3R)BSC499* | 14.0 | 2 +/- 1 | 4 | 24 |
| +/ *Df(3R)BSC739* | 23.9 +/- 0.1 | 36 +/- 6 | 72 | 36 |
| *unc80[GS12792]/ Df(3R)BSC739* | NA | 0 +/- 0 | 0 | 12 |
| *unc80[x42]/* *Df(3R)BSC739* | NA | 0 +/- 0 | 0 | 11 |

*^1^ unc79 and unc80 mutant alleles were backcrossed to* iso31 *for >=6 generations.*
