# Supplementary material for: UNC79 and UNC80, Putative Auxiliary Subunits of the NARROW ABDOMEN Ion Channel, Are Indispensable for Robust Circadian Locomotor Rhythms in Drosophila
Source: PLoS One. 2013 Nov 5;8(11):e78147. doi: 10.1371/journal.pone.0078147 (PMC3818319; doi:10.1371/journal.pone.0078147)
Supplement: Table S2 — DD rhythmicity in unc79 and unc80 single and double heterozygotes. (DOCX) [file pone.0078147.s006.docx]

**TABLE S2. DD rhythmicity in *unc79* and *unc80* single and double heterozygotes.**

| **Genotype ^1^** | **Period (hrs)** | **Power** | **Rhythmic (%)** | **n** |
| --- | --- | --- | --- | --- |
| wild-type males | 24.1 +/- 0.1 | 63 +/- 5 | 100 | 41 |
| *unc79 [x25]/ +* males | 23.9 +/- 0.1 | 64 +/- 6 | 95 | 40 |
| *unc80 [x42]/ +* males | 24.0 +/- 0.1 | 50 +/- 5 | 89 | 38 |
| *unc79 [x25]/ unc80 [X42]* males | 24.0 +/- 0.1 | 56 +/- 4 | 94 | 52 |
| wild-type females | 24.5 +/- 0.1 | 35 +/- 4 | 91 | 35 |
| *na[e04835]/+* females | 24.5 +/- 0.1 | 39 +/- 4 | 73 | 56 |
| *unc79[x25]/+* females | 24.3 +/- 0.1 | 34 +/- 6 | 78 | 23 |
| *unc80[x42]/+* females | 24.9 +/- 0.2 | 21 +/- 6 | 58 | 19 |
| *na[e04835]/+;; unc79[x25]/+* females | 24.5 +/- 0.1 | 40 +/- 8 | 75 | 20 |
| *na[e04835]/+;; unc80[x42]/+* females | 24.2 +/- 0.1 | 30 +/- 8 | 61 | 18 |

*^1^ All strains were backcrossed to* iso31 *for >=6 generations.*
