# Supplementary material for: UNC79 and UNC80, Putative Auxiliary Subunits of the NARROW ABDOMEN Ion Channel, Are Indispensable for Robust Circadian Locomotor Rhythms in Drosophila
Source: PLoS One. 2013 Nov 5;8(11):e78147. doi: 10.1371/journal.pone.0078147 (PMC3818319; doi:10.1371/journal.pone.0078147)
Supplement: Materials and Methods S1 — (DOCX) [file pone.0078147.s007.docx]

**SUPPORTING MATERIALS AND METHODS**

**Quantitative real-time PCR:** For each experiment, total RNA was extracted from 30 adult heads per genotype using TRIzol reagent (Invitrogen). Quantitative real-time polymerase chain reaction (qPCR) was performed using the One Step-Quantitech SYBR-Green RT-PCR Kit (Qiagen) and a LightCycler 480 system (Roche) using 100ng of total RNA and the following primers: 5’-TCATATCCGGCAATGCTG-3’ and 5’-TATAGACGGCATGTACCGGACC-3’ for *na*,  5’-CATCCTCATGCTGGACATAC-3’ and 5’-ACTCTGCCACCTTTAAGGC-3’ for *unc79*, 5’-CGCAAAGATCGGATAAGTG-3’ and 5’-CGGATAAACTTCAGGATGGG-3’ for *unc80*, and 5’-CTGCCCACCGGATTCAAG-3’ and 5’-CGATCTCGCCGCAGTAAAC-3’ for *rp49*. For each biological replicate and primer set, the qPCR assay was carried out in triplicate. Three independent experiments were performed for each mutant strain (*na^e04385^, unc79^x25^*, and *unc80^x42^*) and the corresponding isogenized control strains. Samples were normalized to *rp49* and the differences in expression between mutant and control strains were calculated using the ΔΔCt method [[1](#_ENREF_1)]. Significance was determined by Student’s T-test.

**REFERENCE**

1. Livak KJ, Schmittgen TD (2001) Analysis of relative gene expression data using real-time quantitative PCR and the 2(-Delta Delta C(T)) Method. Methods 25: 402-408.
